# Subclinical exposure to *Streptococcus pyogenes* drives the development of long-lived homologous immunity

**DOI:** 10.1038/s41467-026-76176-1

**Published:** 2026-08-12

**Authors:** Ailin Lepletier, Despena Vedis, Victoria Ozberk, Merrina Anugraham, Darrell Bessette, Christie Short, Ainslie Calcutt, Hannah R. Frost, Kristy I. Azzopardi, Andrew C. Steer, Daniel Kolarich, Joshua Osowicki, Michael F. Good, Manisha Pandey

**Affiliations:** 1 Institute for Biomedicine and Glycomics, Griffith University, Gold Coast, QLD Australia; 2 School of Science & Technology, University of New England, Armidale, NSW Australia; 3 Tropical Diseases Research Group, Murdoch Children’s Research Institute, Melbourne, VIC Australia; 4 Department of Paediatrics, University of Melbourne, Melbourne, VIC Australia; 5 Department of Infectious Diseases, Royal Children’s Hospital Melbourne, Melbourne, VIC Australia; 6 School of Environment and Science, Griffith University, Gold Coast, QLD Australia

**Keywords:** Bacterial infection, Bacteriology, Antibodies, Pathogens

## Abstract

Immunity to *Streptococcus pyogenes* infection appears to develop progressively through repeated exposure during early life. However, the intensity or duration of exposure required is unknown, as to why some individuals appear to develop immunity, despite having few or no previously detected infections. Here, drawing on samples from a human model of pharyngeal *S. pyogenes* infection, we investigate whether symptomatic disease is required for induction of protective immune responses. We show that challenge with M75 *S. pyogenes* elicits M75-specific serum antibodies and memory B cells in both symptomatic and asymptomatic participants, with responses persisting for at least 6 months. M75-specific IgG from asymptomatic participants exhibit altered Fc glycosylation traits post-challenge, associated with enhanced binding to M75 *S. pyogenes* and protection when transferred into a murine model of respiratory tract infection. These findings demonstrate that asymptomatic exposure to M75 *S. pyogenes* contributes to the development of streptococcal immunity that emerges by adulthood.

## Abstract (web summary)

The rate of infection with *Streptococcus pyogenes* reduces with age and is thought to be driven at least in part due to immunity derived from repeated exposure during life. Here the authors using data from human challenge studies and animal models of infection, suggest subclinical exposure to *S.pyogenes* results in long lived homologous immunity.

## Introduction

*Streptococcus pyogenes* (Group A *Streptococcus*, Strep A) is a leading human-restricted pathogen with a persistent and profound global disease burden. It causes a broad clinical spectrum of illness, spanning superficial through to severe infections and post-infectious syndromes, including rheumatic heart disease^1,2^. Every year, up to a billion people are affected, and more than 500,000 deaths are attributable to *S. pyogenes*^3^. ‘Strep throat’ or *S. pyogenes* pharyngitis is a ubiquitous childhood illness, with incidence peaking during the primary school years^4,5^. Lower incidence in adults has generally been ascribed to immunity accumulated through repeated exposure in childhood to different *S. pyogenes* strains^6^. Although the precise mechanisms of immunity against *S. pyogenes* remain uncertain, several humoral and cellular responses have been strongly implicated in protection against colonization or disease^7^. These responses include antibodies that bind to bacteria, enabling neutrophils and monocytes at the site of infection to recognize and eliminate them through opsonophagocytosis^7^. In individuals resistant to pharyngeal colonization, such antibodies can also prevent early bacterial establishment by blocking adherence and promoting rapid bacterial clearance^8^.

Allelic polymorphism of the *S. pyogenes* M-protein is a major impediment to the development of immunity. To date, over 250 distinct serotypes have been identified based on their amino-terminal hypervariable region (HVR)^9^. Longitudinal studies indicate that naturally acquired antibodies against the M-protein can provide homologous protection but offer limited cross-protection against disease caused by heterologous strains^10,11^. While the M-cluster concept explains some degree of cross-protective immunity^12^, it remains unclear how most children develop protective immunity by their second decade of life^12^, considering that only around 15% of young children experience symptomatic *S. pyogenes* pharyngitis in any given year^1^. A potential explanation is that repeated exposures boost immunity to antigens that are broadly conserved across strains, complementing narrower responses to type-specific antigens such as the M-protein HVR. We previously showed that antibodies to the conserved C-repeat region promote immune-mediated killing of *S. pyogenes* irrespective of its M-protein serotype^13–15^. However, C-repeat-specific immunity is unlikely to account for naturally acquired immunity because this region is poorly immunogenic (‘cryptic’) during both experimental and natural infections. Antibodies to this region do not develop in children from highly streptococcal-endemic areas until well into their second decade of life^16^ nor do they arise following repeated exposures of mice to *S. pyogenes*^17^.

Given the high prevalence of asymptomatic pharyngeal *S. pyogenes* colonization or ‘carriage’ in 10–20% of school-age children^18^, and the circulation of multiple serotypes within communities at any given time^19^, it is reasonable to hypothesize that repeated asymptomatic (subclinical) exposures shape protective immune responses to *S. pyogenes*. Here, we explore this hypothesis in a well-characterized cohort of participants from the CHIVAS-M75 *S. pyogenes* human pharyngeal challenge study^20–22^. While acute symptomatic pharyngitis following direct tonsillar inoculation was the most common outcome, a subset remained asymptomatic despite lacking significant pre-existing serotype-specific antibodies to the M75 strain, enabling us to investigate the immunological outcomes of subclinical infections. At 6 months post-challenge, increased levels of memory B cells and antibodies targeting the M75 hypervariable region (HVR), but not a conserved cryptic M-protein epitope (p*17), were observed in participants both with and without pharyngitis. Using several approaches to assess antibody function, including bacterial binding, IgG Fc glycosylation traits, and passive antibody transfer in a murine model of respiratory tract infection, we show that subclinical exposures can elicit immune responses that contribute to long-term protection against *S. pyogenes*.

## Results

### Murine antibodies against the M75 hypervariable region bind specifically to M75 *S. pyogenes*

Our initial studies aimed to characterize humoral immune responses elicited by the M-protein peptides, p*17 and M75-HVR (Supplementary Fig. 1A). Although p*17 is poorly recognized following infection of mice, it is known that antibodies induced against the p*17-based vaccine can protect against infection^23–26^. BALB/c mice were immunized with either p*17-diphtheria toxoid (DT) or M75-HVR-DT (M75-DT), each adjuvanted with aluminium hydroxide (Alum), following a 3-dose schedule as in a Phase 1 clinical trial^27^. Sera from both groups were collected two weeks after the final vaccine dose and analyzed by indirect ELISA and flow cytometry-based binding assays to assess IgG antibody responses against p*17 and M75-HVR peptides, as well as against live *S. pyogenes* bacteria (Supplementary Fig. 1B), respectively. Serum from M75-DT immunized mice specifically recognized the M75 peptide (Supplementary Fig. 1C), whereas serum from p*17-DT-immunized mice exclusively recognized p*17 (Supplementary Fig. 1D). Using flow cytometry, we demonstrated that serum from M75-DT–immunized mice specifically bound to M75 *S. pyogenes*, with no detectable reactivity to an M1 strain (Supplementary Fig. 1E). In contrast, serum from p*17-DT–immunized mice recognized both M75 and M1 strains (Supplementary Fig. 1F), consistent with p*17 being a conserved epitope across *S. pyogenes* strains.

### Human pharyngeal challenge with the M75 *S. pyogenes* elicits memory responses directed against the M-protein hypervariable region

To assess the long-term human immune responses to infection, we examined memory B cell populations and serum antibodies from CHIVAS-M75 participants^20–22^. Antigen-specific responses to p*17 and M75-HVR were assessed for 13 participants who developed acute symptomatic pharyngitis following pharyngeal challenge with M75 *S. pyogenes*. These were compared to responses from 5 participants who remained asymptomatic (non-pharyngitis) and had no detectable M75 *S. pyogenes* by culture or qPCR from throat swabs collected twice daily from 24 h after challenge until discharge. Systemic immune responses were longitudinally analyzed at pre-challenge, 1 month, and 6 months post-challenge.

**Fig. 1:**
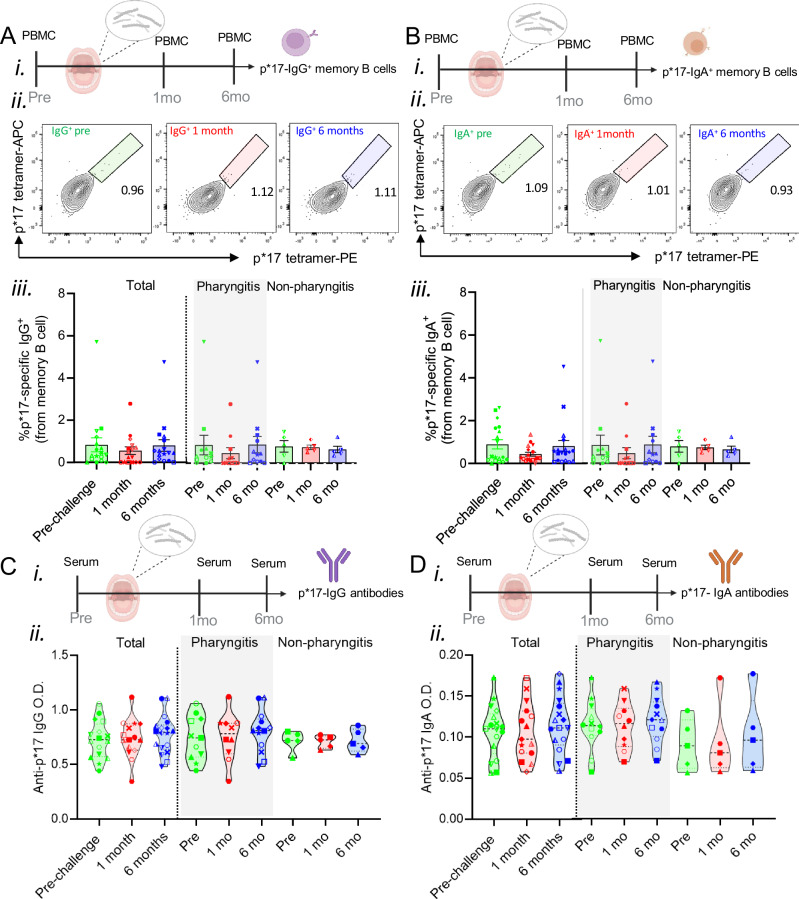
Memory responses to p*17 following human infection with *S. pyogenes* M75. Created in BioRender. Pandey, M. (2026) https://BioRender.com/3viurh1. Flow cytometry analysis of p*17-specific **A** IgG^+^ and **B** IgA^+^ memory B cells in the peripheral blood of participants in a controlled human infection study. i. Schematic overview of study timeline and collection of peripheral blood mononuclear cells (PBMC) for analysis of B cells in the peripheral blood. ii. Flow cytometry gating strategy for analysis of antigen-specific memory B cells in the peripheral blood. Representative plots are shown from a single participant across different time points. iii. The percentage of p*17-specific memory B cells is shown in the bar graphs at pre-challenge (day −1), 1 month, and 6 months post-challenge with mean +/- SEM. ELISA was used to detect anti-p*17-specific **C** IgG and **D** IgA antibodies in PBMC-matched serum samples. i. Schematic overview of study timeline and collection of serum for analysis of antibodies. ii. Violin plots show O.D. at 450 nm absorbance values for sera collected at pre-challenge (day −1) and 6 months post-challenge, using a 1:20 dilution. The center line indicates the median and the box represents the interquartile range. For both tetramer and ELISA analyses, data are stratified by participants with and without pharyngitis (total *n* = 18; pharyngitis, *n* = 13; non pharyngitis, *n* = 5). Each symbol represents an individual participant. Statistical analysis was performed using one-way ANOVA with Tukey’s post-hoc test. Source data are provided as a Source Data file.

To evaluate immunity against *S. pyogenes* and explore the dynamics of antigen-specific memory B cells and antibody responses post-challenge, we generated fluorescent antigen-tetramers and analyzed responses to p*17 and the type-specific HVR region of the M75 protein. To determine whether a single M75 *S. pyogenes* infection induced memory B cells recognizing the conserved region of the M-protein, we specifically assessed responses to p*17 (Fig. 1A, B). Flow cytometry-based tetramer analysis was used to characterize p*17-specific memory B cells (CD27^+^CD21^+^) within both the IgA and IgG subclasses (Supplementary Fig. 2A). The frequency of p*17-specific IgG^+^ memory B cells remained low and unchanged following pharyngeal challenge with M75 *S. pyogenes* out to 6 months in both symptomatic and asymptomatic participants (Fig. 1A). Among participants who developed symptomatic disease following challenge, there was a trend toward increased p*17-specific IgA⁺ at 6 months compared with 1 month (mean difference (Δ) 0.5528%; 95% CI −0.2207 to 1.326, *P* > 0.05) (Fig. 1B). Antibody response analyses in the same participants similarly revealed that IgG antibody levels against p*17 remained low and unchanged across all time points in all participants following pharyngeal challenge (Fig. 1C). Serum IgA responses to the p*17 antigen were minimal (O.D. <0.2 at a dilution of 1:20); however, a trend toward increased p*17-specific serum IgA responses was observed at 6 months compared with pre-challenge (Δ 0.0067; − 0.001199 to 0.01220, *P *> 0.05) in participants who developed pharyngitis (Fig. 1D).

**Fig. 2:**
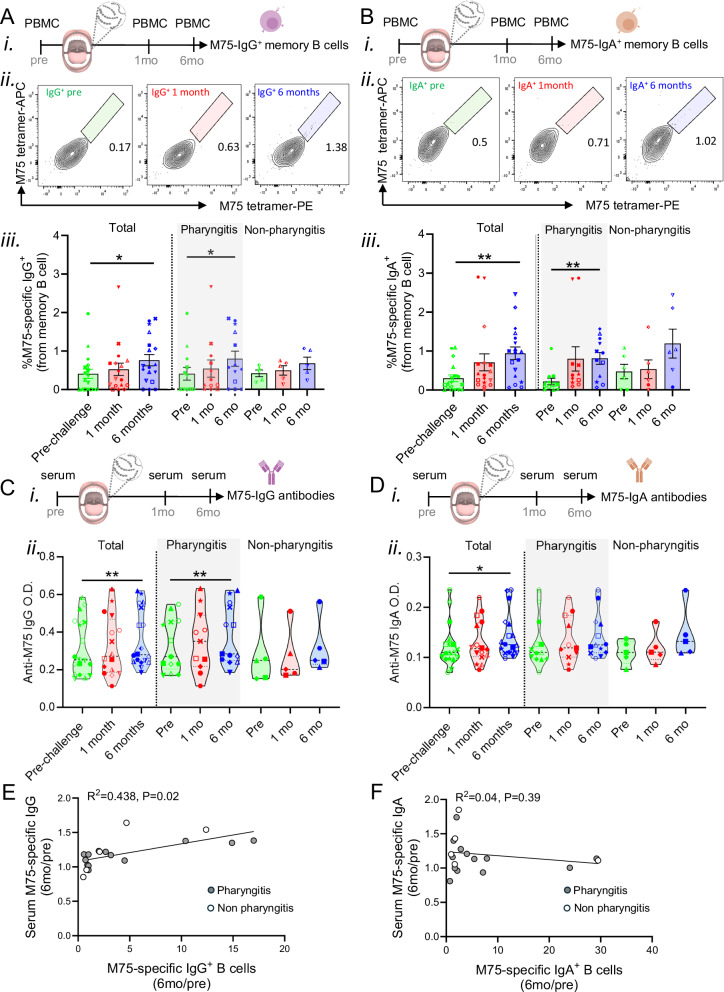
Memory responses to M75-HVR following human infection with *S. pyogenes* M75. Created in BioRender. Pandey, M. (2026) https://BioRender.com/44cb3zo. Flow cytometry analysis of M75-specific **A** IgG^+^ and **B** IgA^+^ memory cells in the peripheral blood of participants in a controlled human infection study. i. Schematic overview of study timeline and collection of PBMC for analysis of B cells. ii. Flow cytometry gating strategy for analysis of antigen-specific memory B cells in the peripheral blood. Representative plots are shown from a single participant across different time points. iii. The percentage of M75-specific memory B cells is shown in the bar graphs at pre-challenge (day −1), 1 month, and 6 months post-challenge, with mean +/- SEM. ELISA was used to detect anti-M75-specific **C** IgG and **D** IgA antibodies in PBMC-matched serum samples. i. Schematic overview of study timeline and collection of serum for analysis of antibodies. ii. Violin plots show at O.D. 450 nm absorbance values for sera collected at pre-challenge (day −1), 1 month and 6 months post-challenge, using a 1:20 dilution. The center line indicates the median and the box represents the interquartile range. For both tetramer and ELISA analyses, data are stratified by participants with and without pharyngitis (total *n* = 18; pharyngitis, *n* = 13; non pharyngitis, *n* = 5). Each symbol represents an individual participant. Statistical analysis comparing different time points within each group was performed using one-way ANOVA with Tukey’s post-hoc test. *P < 0.05, **P < 0.01. Correlation between the fold change in M75-specific (**E**) IgG+ and (**F**) IgA+ memory B cells and the fold change in serum M75-specific antibodies at 6 months post- vs pre-challenge. Full circles = pharyngitis, empty circles = non-pharyngitis participants. Correlation coefficient and *P* value were determined using two-sided Spearman’s method with a linear regression line included to indicate linear trends. Source data are provided as a Source Data file.

**Fig. 3:**
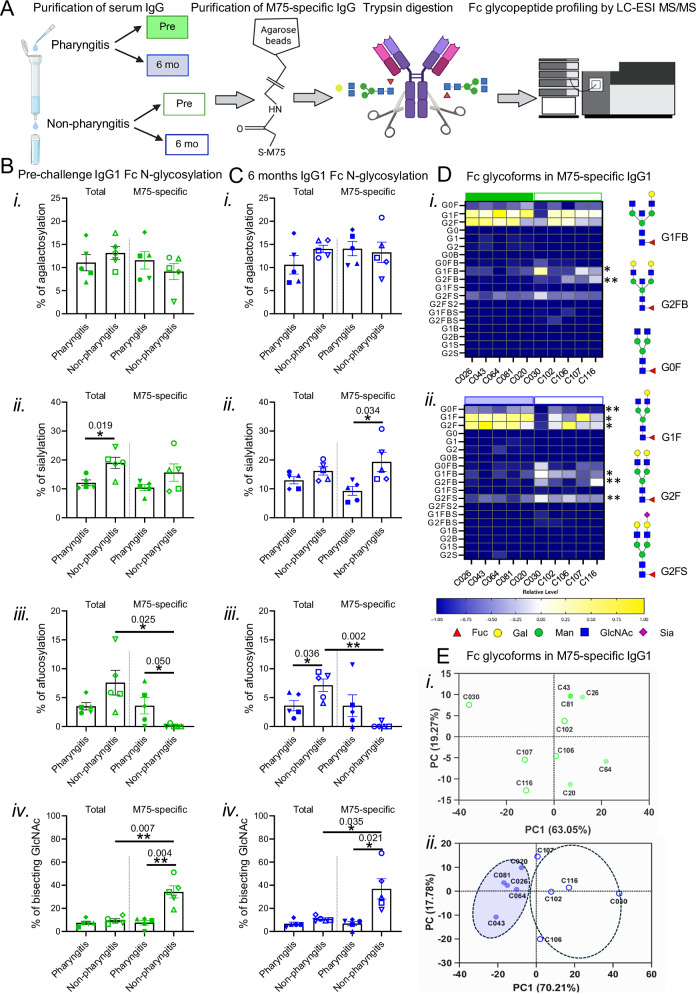
Comparative profiling of IgG Fc N-glycosylation in total and M75-specific serum IgG1 from participants with and without pharyngitis. Created in BioRender. Pandey, M. (2026) https://BioRender.com/6f6n25d. **A** Schematic overview of M75-specific IgG purification, digestion and Fc glycan profiling by liquid chromatography tandem mass spectrometry (LC-MS/MS). Bar graphs depict the relative abundance of glycans in total and M75-specific IgG1 antibodies at **B** baseline (pre-challenge) and **C** 6 months post-challenge with mean +/- SEM (*n* = 5/group). Data represent individual unpaired samples from participants with (closed symbols) and without (open symbols) pharyngitis collected at pre-challenge (green) and 6 months-post challenge (blue) (*n* = 5/group). Data are shown as the average of two technical replicates per participant. Unpaired *t*-test between participants with and without pharyngitis was used for statistical analysis, while paired *t*-test was used for within group comparisons between total and M75-specific IgG1. *P < 0.05, **P < 0.01. **D** Heatmaps illustrate the fractional distribution of individual Fc glycoforms in M75-specific IgG1 antibodies at i. pre-challenge and ii. 6 months post-challenge. A schematic representation highlights the Fc glycoforms attached to IgG1 that are differentially distributed between participants with and without pharyngitis across the different time points. Unpaired *t*-test between participants with and without pharyngitis was used for statistical analysis. All statistical tests were two-sided. *P < 0.05, **P < 0.01. **E** Principal component analysis (PRISM version 9) shows the relationship of Fc glycoforms data from 5 biological replicates across participants with and without pharyngitis collected at i. pre-challenge (green) and ii. 6 months-post challenge (blue). Source data are provided as a Source Data file.

In those who developed symptomatic disease post-challenge, the frequency of M75-specific IgG^+^ memory B cells increased significantly at 6 months (mean difference (Δ) 0.4633; 95% CI 0.03542 to 0.8912, *P < 0.05) (Fig. 2A). M75-specific IgA^+^ memory B cells showed an early increase, observed at 1 month (Δ 0.5328; −0.03110 to 1.097, *P* > 0.05), which also endured for at least 6 months post-challenge (Δ 0.5577; 0.2452 to 0.8702, **P < 0.01) (Fig. 2B). In asymptomatic participants, there was a trend toward an increase at 6 months for both M75-specific IgG^+^ (Δ 0.2600; −0.2548 to 0.7748, *P* > 0.05) (Fig. 3A) and IgA^+^ (Δ 0.8560; −0.4091 to 2.121, *P* > 0.05), Fig. 2B) memory B cells.

Similarly, antibody analysis showed a significant rise in M75-specific IgG levels in participants with pharyngitis but was detected only at 6 months post-challenge (Δ 0.05202; 0.02262 to 0.08143, **P < 0.01) (Fig. 2C). This increase was driven specifically by the M75-specific IgG3 subtype (Δ 0.03722; 0.0004340 to 0.07400, *P < 0.05) (Supplementary Fig. 1B) with no significant changes in IgG1, IgG2, or IgG4 levels (Supplementary Fig. 1C–E). In those participants, M75-specific serum IgA antibodies also showed an increase at 6 months post-challenge (Δ 0.01366; −0.002654 to 0.02998, *P* > 0.05) (Fig. 2D). Although a significant increase in IgG antibodies was observed only in symptomatic participants, asymptomatic individuals showed a comparable mean change in M75-specific IgG (Δ 0.03550; −0.04207 to 0.1131, *P* > 0.05) and IgG3 (Δ 0.03512; −0.05865 to 0.1289, *P* > 0.05) levels at 6 months post-challenge. Mean antibody levels at this time point were also similar between asymptomatic participants and those with pharyngitis (Supplementary Fig. 2F). The increase in IgG^+^ memory B cells binding the M75 antigen correlated with increased M75-specific serum IgG at 6 months relative to pre-challenge (R^2^ = 0.438, *P* = 0.02, Fig. 2E). Interestingly, the increase in IgA^+^ memory B cells binding the M75 *S. pyogenes* antigen did not correlate with increased M75-specific serum IgA (R^2^ = 0.04, *P* = 0.39, Fig. 2F). There was no significant difference in pre-challenge M75-specific memory B cells and antibodies between participants who did and did not develop pharyngitis following challenge (Fig. 2C, D).

In summary, exposure to M75 *S. pyogenes* induces long-term, strain-specific systemic immunity, reflected by sustained serum Ig levels and memory B cell responses involving both IgG and IgA. Although evident in both groups of participants, the immune responses were more pronounced in those who developed clinical disease.

### **IgG Fc glycosylation is associated with clinical manifestations of*S. pyogenes*infections**

Post-translational modifications, particularly glycosylation of the constant (Fc) IgG domain, are well-known features of all IgGs and play a critical role in modulating antibody effector functions, including complement activation and Fc receptor engagement^28,29^.

We initially assessed whether pre-challenge IgG Fc N-glycosylation traits could be a distinguishing factor between participants with and without pharyngitis. We purified total (entire IgG pool present in plasma) and M75-specific IgG from asymptomatic and symptomatic participants prior to challenge and at 6 months post-challenge. Individuals with high pre-challenge levels of anti-M75 IgG antibodies were excluded from the CHIVAS-M75 trial^21^; however, affinity purification enabled us to enrich these antibodies from the participants (Figs. 2C and 3A). Mass spectrometry-based characterization revealed that IgG1 antibodies were the major IgG subclass targeting the M75-epitope across all participants. Participants who remained asymptomatic following challenge exhibited a distinct Fc glycosylation profile for total IgG1 compared to participants who became symptomatic. Prior to challenge, asymptomatic participants displayed higher level of sialylated glycans (1.5-fold increase, *P < 0.05, Fig. 3Bii). At 6 months post-challenge, they showed increased levels of afucosylated glycans (2-fold increase, *P < 0.05, Fig. 3Cii). More pronounced differences were observed in the Fc glycosylation profile of M75-specific IgG1 antibodies from asymptomatic participants. At pre-challenge, this was characterized by a marked reduction in afucosylation (30-fold decrease, *P < 0.05; Fig. 3Biii) and a 4.9-fold increase in bisecting GlcNAc moieties (**P < 0.01; Fig. 3Biv). The increase in bisecting GlcNAc was driven by elevated levels of G1FB (3.1-fold increase, *P < 0.05) and G2FB N-glycans (13.9-fold increase, **P < 0.01; Fig. 3Di).

Notably, differences in Fc glycosylation between total IgG1 and M75-specific IgG1 were observed exclusively in participants who remained asymptomatic. M75-specific IgG1 purified prior to challenge exhibited significantly reduced afucosylation (17.5-fold decrease, *P < 0.05; Fig. 3Biii) and an increased proportion of bisecting GlcNAc (3.9-fold increase, **P < 0.01; Fig. 3Biv) compared with total IgG1. This pattern was maintained at 6 months post-challenge, with persistent reductions in afucosylation (34-fold decrease, ***P < 0.005; Fig. 3Ciii) and increased bisecting GlcNAc (3.5-fold increase, *P < 0.05; Fig. 3Civ). These differences were driven by specific glycoform alterations in those who remained asymptomatic, including increased levels of G2FS N-glycans (2.1-fold increase, ***P < 0.01), together with reduced levels of G0F (2.5-fold decrease, **P < 0.01), G1F (1.8-fold decrease, *P < 0.05), and G2F (1.6-fold decrease, *P < 0.05) compared with symptomatic individuals (Fig. 3Di, ii). Consistent with these findings, principal component analysis demonstrated that disease manifestation shaped M75-specific IgG1 Fc glycosylation at 6 months post-challenge, with symptomatic and asymptomatic participants segregating into two well-defined glycoform clusters (Fig. 3Eii).

**Fig. 4:**
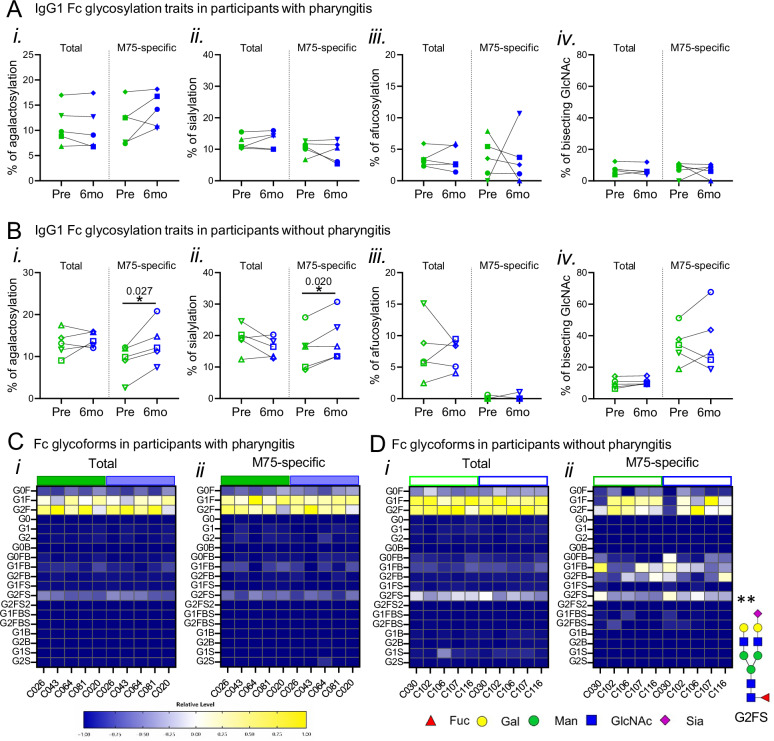
Analysis of Fc N-glycosylation profile of total and M75-specific serum IgG1 antibodies at pre- and 6 months post-challenge. Line graphs depict the relative abundance of glycans in total and M75-specific IgG1 antibodies in participants who (**A**) developed pharyngitis or (**B**) remained asymptomatic. Heatmaps illustrate the fractional distribution of individual Fc glycoforms in (**C**) total and **D** M75-specific IgG1 antibodies. Data represent paired samples collected pre-challenge (green) and 6 months post-challenge (blue) from participants with and without pharyngitis (*n* = 5/group). Two-sided paired *t*-test between pre- and post-challenge was used for statistical analysis *P < 0.05, **P < 0.01. Data from each participant are presented as the average of two technical replicates. Source data are provided as a Source Data file.

We further analyzed changes at 6 months post-challenge relative to pre-challenge levels separately in participants with and without pharyngitis. In those who manifested clinical disease, no significant differences were detected in overall IgG Fc N-glycosylation traits (Fig. 4A). No changes in IgG1 Fc N-glycosylation were observed in total IgG1 purified from asymptomatic participants at 6 months post-challenge compared to pre-challenge levels either (Fig. 4B). However, at 6 months, M75-specific IgG1 showed a significant increase in agalactosylation (1.5-fold, *P < 0.05) and sialylation (1.3-fold, *P < 0.05, Fig. 4Bi, ii). The glycoform compositions of total and M75-specific IgG1 antibodies remained unchanged in participants with pharyngitis (Fig. 4C). In contrast, asymptomatic individuals demonstrated a selective enrichment of G2FS glycoforms (1.3-fold, **P < 0.01, Fig. 4D) relative to pre-challenge.

Collectively, these findings indicate that Fc glycosylation features of M75-specific IgG1 antibodies present prior to challenge are associated with susceptibility to symptomatic disease following M75 *S. pyogenes* challenge. Moreover, the post-challenge glycosylation remodeling observed selectively in asymptomatic participants suggests a qualitative enhancement of M75-specific IgG1 antibody effector function, occurring in the absence of substantial changes in overall systemic antibody levels.

### Pharyngeal challenge with M75 *S. pyogenes* induces durable strain-specific IgG antibodies with minimal to no cross-reactivity to heterologous isolates

**Fig. 5:**
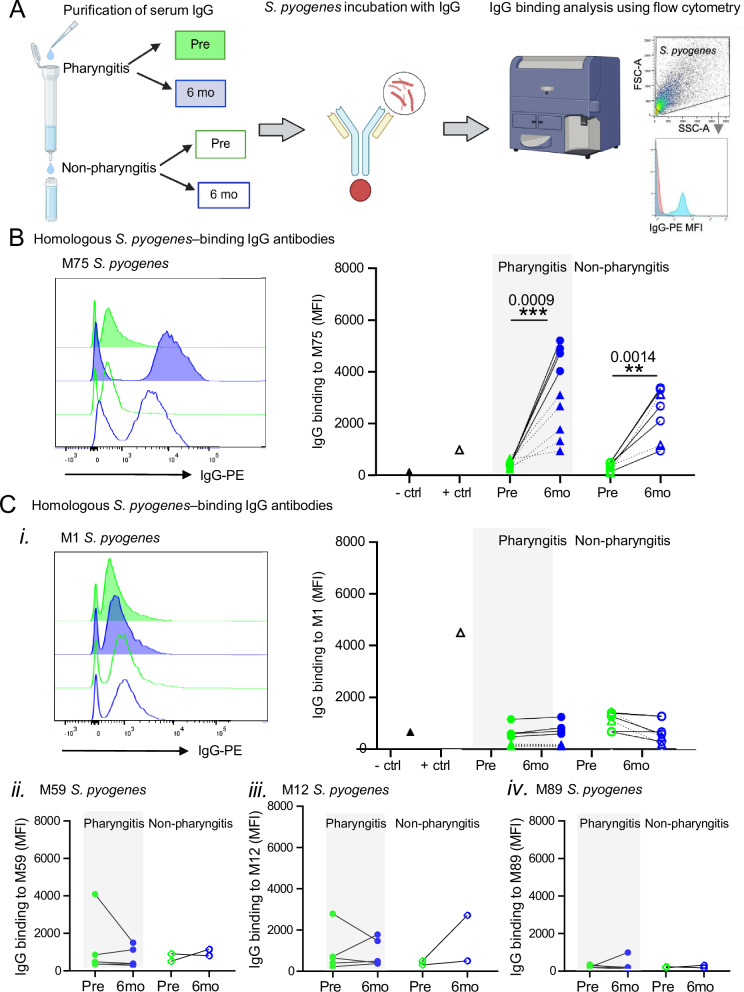
Binding of serum IgG to homologous and heterologous *S. pyogenes* strains. Created in BioRender. Pandey, M. (2026) https://BioRender.com/ffq6g72. **A** Schematic overview of IgG purification from serum and flow cytometry assessment of IgG binding to *S. pyogenes*. Representative histograms and line graphs illustrate IgG binding responses from participants with pharyngitis, P, and non-pharyngitis, NP, to (**B**) homologous and **C** heterologous strains. For M75 and i M1 binding assessment, intravenous immunoglobulin (IVIg) was included as a positive control, while normal human IgG pre-adsorbed against M1 and M75 bacteria served as the negative control. Binding was assessed in two independent experiments, with Experiment 1 represented by circles and Experiment 2 by triangles. Five distinct IgG samples from participants with pharyngitis were included in each experiment (total *n* = 9/group). For non-pharyngitis participants, five samples were analyzed in the first experiment, and two of these were repeated in the second experiment. IgG binding to ii M59, iii pM12, and iv M89 was assessed in a single experiment (*n* = 5/ group). For all conditions, samples were collected at pre-challenge (green) and 6 months post-challenge (blue) from participants with (closed symbol) and without (open symbol) pharyngitis. Each symbol represents one participant. The level of binding of purified IgG antibodies to *S. pyogenes* is shown as mean fluorescence intensity (MFI). One-way ANOVA followed by Bonferroni’s comparisons tests was used to assess differences in IgG binding among trial participants in all statistical analyses. **P < 0.01, ***P < 0.001. Source data are provided as a Source Data file.

The temporal changes and differences observed in the IgG Fc N-glycosylation of symptomatic and asymptomatic participants prompted us to examine their functional properties more closely. To increase the sensitivity of our analyses, we purified total IgG and used flow cytometry to assess binding to homologous and heterologous *S. pyogenes* strains at both pre-and 6 months post-challenge in participants with and without pharyngitis (Fig. 5A). Negative controls were generated by absorbing normal human IgG against relevant *S. pyogenes* strains, and binding was interpreted relative to these controls.

For the homologous M75 strain, the negative control mean fluorescence intensity (MFI) was 129, and pre-challenge IgG MFIs ranged from 110 to 664, indicating low-level baseline binding (Fig. 5B). Following challenge, binding of purified IgG to the M75 *S. pyogenes* ranged from 935 to 5212 MFI and increased significantly at 6 months post-challenge in participants with symptomatic pharyngitis relative to their pre-challenge levels (12.3-fold, ***P < 0.005, Fig. 5B). A similar increase was also evident in asymptomatic participants (6.5-fold, **P < 0.01, Fig. 5B).

For M1, the negative control MFI was 660, and pre-challenge IgG MFIs ranged from 104 to 1430, consistent with binding that was comparable to, or only marginally above, background levels (Fig. 5Ci). Following challenge, M1 MFIs ranged from 105 to 1266. In line with these observations, and similar to sera from mice immunized with M75-DT (Supplementary Fig. 1E), IgG from CHIVAS trial participants at 6 months post-challenge, irrespective of pharyngitis status, showed minimal binding to heterologous M1 *S. pyogenes* (Fig. 5Ci). Supporting strain-specific IgG responses, binding to additional heterologous strains (M12, M59, and M89) was also assessed and showed no increases following challenge (Fig. 5Cii–iv). In some cases, relatively high pre-challenge binding to M59 and M12 was observed (>2000 MFI, Fig. 5Cii, iii), indicating the presence of antibodies prior to infection to heterologous *S. pyogenes* strains in some individuals.

### Subclinical exposure to *S. pyogenes* induces long-term, functionally protective IgG immunity

**Fig. 6:**
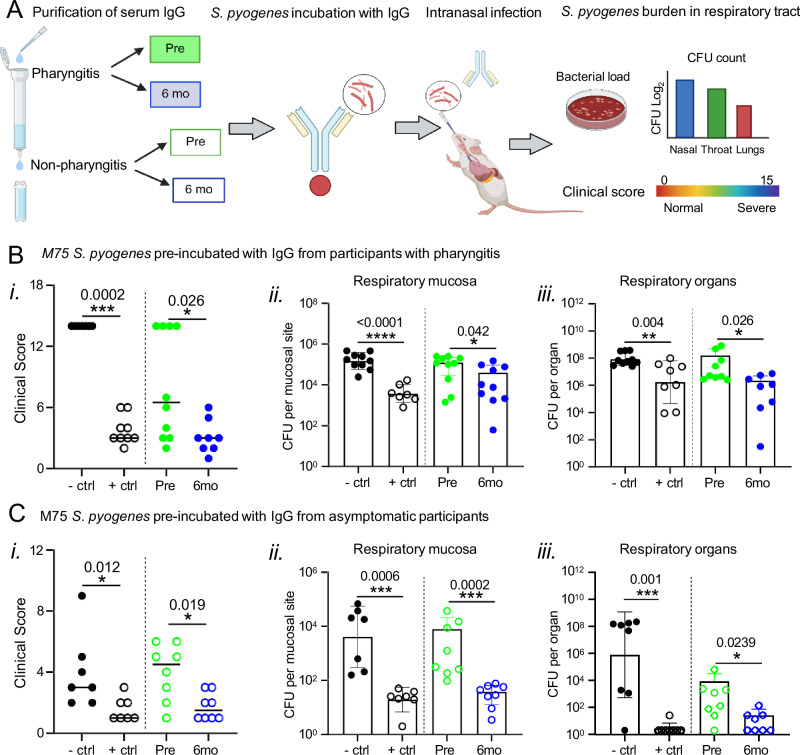
Functional analysis of purified serum IgG following *S. pyogenes* infection. Created in BioRender. Pandey, M. (2026) https://BioRender.com/r4azl37. **A** Schematic overview of serum IgG purification and assessment of efficacy following intranasal inoculation with IgG-pre-incubated *S. pyogenes*. SCID mice (were inoculated intranasally with M75 *S. pyogenes* pre-incubated with pooled IgG from **B** symptomatic participants (*n* = 10/group) or **C** asymptomatic participants (control, *n* = 7/group; test *n* = 8/group) at pre-challenge and 6 months post-challenge. IgG purified from rat sera immunized with p*17-K4S2-CRM/Alum or left as naïve was used as a control. i. Graph shows clinical score of mice on day 2 following inoculation. ii, iii *S. pyogenes* colony-forming units (CFU) were enumerated in samples collected from ii respiratory mucosal sites (throat swabs and nasal shedding) and iii respiratory organs (lungs and nasal-associated lymphoid tissue [NALT]), on day 2 post-challenge. Data are represented as geometric mean ± geometric SD. A two-sided unpaired *t*-test was performed to compare weight loss and bacterial burden between mice treated with IgG purified from vaccinated versus naïve rats and from humans at 6 months post- challenge versus pre-challenge. *P < 0.05; **P < 0.01; ***P < 0.001, ****P < 0.0001. Source data are provided as a Source Data file.

We utilized a recently developed in vivo passive transfer model to test IgG-mediated functional immunity. In this assay, M75 *S. pyogenes* was incubated with serum IgG antibodies (‘pre-opsonized’) and then inoculated intranasally into immunocompromised SCID mice, which lack B cell and T cells. Clinical scores (as a general measure of disease severity in *S. pyogenes* -infected mice^24^), and bacterial burden from the entire respiratory tract, stratified by mucosal sites (throat swabs and nasal shedding) and respiratory organs/tissue (lungs and nasal-associated lymphoid tissue [NALT]), were subsequently assessed to determine the protective efficacy of the antibodies (Fig. 6A). We purified IgG from 5 symptomatic and the 5 asymptomatic participants at pre and  6 months post challenge, pooled the IgG at each time point, and tested it against M75 *S. pyogenes* in SCID mice (Fig. 6B, C). In both experiments, controls included IgG purified from rats vaccinated with p*17-K4S2-CRM^26^ or IgG from naïve rats. Mice inoculated with *S. pyogenes* pre-incubated with IgG collected 6 months post-challenge exhibited significantly lower clinical scores compared with mice receiving bacteria pre-incubated with pre-challenge IgG. This protective effect was observed for IgG from both symptomatic (*P < 0.05, Fig. 6Bi) and asymptomatic participants (*P < 0.05, Fig. 6Ci).

In mice receiving IgG from symptomatic participants, this was associated with a 72% reduction in *S. pyogenes* colonization of the respiratory mucosa (*P < 0.05) (Fig. 6Bii) and a > 98% reduction in respiratory organs/tissue colonization (*P < 0.05) (Fig. 6Biii). Similarly, IgG from asymptomatic participants resulted in a > 99% of reduction in mucosal colonization (***P < 0.001) and 71% (*P < 0.05) in respiratory organ colonization (Fig. 6Cii, iii, respectively). There were no significant differences observed between IgG from naïve rats and the human pre-challenge IgG (Fig. 6B, C). Across all mucosal sites analyzed for bacterial burden, IgG from vaccinated rats resulted in 97% and 99% reduction (****P < 0.001 and ***P < 0.001; Fig. 6Bii, Cii, respectively). Similarly, passive transfer of IgG from vaccinated rats resulted in reductions in bacterial burden within the respiratory tract, with decreases of 85% and 99% observed (**P < 0.01 and ***P < 0.001; Fig. 6Biii, Ciii, respectively). Notably, the reduction in bacterial burden at mucosal sites achieved with post-challenge IgG from asymptomatic participants was comparable to that observed with IgG from vaccinated rats relative to naïve rats (>99.9% reduction, ***P < 0.005). In contrast, IgG from symptomatic participant appeared more effective at limiting bacterial burden within respiratory organs/tissues (>98% reduction, *P < 0.05).

Taken together, serum IgG antibodies induced by subclinical *S. pyogenes* infection exhibit robust functional activity, comparable to that of symptomatic infection, and confer protection against both subsequent mucosal and severe/invasive infections.

## Discussion

This study demonstrates that functional immunity to *S. pyogenes* can develop following asymptomatic exposure. Pharyngeal human challenge with M75 *S. pyogenes* elicited class-switched B cells and antibodies (IgG and IgA) in the peripheral blood targeting the type-specific M-protein HVR region. Participants with higher frequency of M75-specific IgG memory B cells at 6 months after challenge also had higher levels of anti-M75 IgG antibodies, suggesting that memory B cells may help maintain or increase IgG antibody levels over time. Symptomatic participants showed a sustained increase in M75-specific IgG⁺ memory B cells and IgG3 antibodies at 6 months, while IgA⁺ memory B cells increased earlier and were maintained over time. Although the absolute changes were modest, the confidence intervals indicate measurable antigen-specific responses. Asymptomatic participants showed changes of similar direction and magnitude, but with wide confidence intervals overlapping zero, likely reflecting limited sample size rather than absence of effect. While the immune responses in asymptomatic participants were lower than those observed in symptomatic individuals, changes in M75-specific IgG1 Fc N-glycosylation traits consistent with enhanced effector function were detected exclusively in the asymptomatic group. Post-challenge IgG mediated homologous binding and bacterial clearance in a murine passive-transfer infection model irrespective of clinical manifestation, whereas pre-challenge IgG did not. Collectively, these results support the hypothesis that immunity to *S. pyogenes* is gradually acquired throughout childhood by repeated asymptomatic (or subclinical) exposures to circulating strains and not exclusively as a consequence of symptomatic infections.

Recent longitudinal studies have also highlighted that asymptomatic *S. pyogenes* exposures may be immunologically significant (*i.e.* capable of triggering measurable immune responses), challenging the long-held assumption that only symptomatic infections contribute meaningfully to immunity. Studies in the USA by Hysmith et al.^30^ and in The Gambia by Keeley et al.^31^ each demonstrated antibody binding responses to M- and non-M-protein antigens following asymptomatic episodes of *S. pyogenes* colonization. This observation is consistent with other findings showing that a significant proportion of people with asymptomatic colonization of the upper respiratory tract develop functional anti-M-protein antibodies^32,33^. Notably, a significant fraction of the children assessed in these studies only developed M-type specific serum IgG antibodies between 6 weeks and a year from detection of a positive throat culture^30,32^, which aligns with our data showing a delayed immune response following pharyngeal challenge. In the 2-year study by Hysmith et al.^30^, new *S. pyogenes* acquisitions did not significantly boost homologous M-type-specific antibody responses. Therefore, it is possible that pre-screening and exclusion of participants with high serum type-specific M75 IgG in the CHIVAS-M75 study^21,22^ may have contributed to the observed immunologically significant M75-specific responses following pharyngeal challenge.

Serum antibodies against the M-protein facilitate the opsonisation and killing of *S. pyogenes* and are believed to contribute to immunity against homologous strains. In the present study, we showed that human serum IgG antibodies purified 6 months post-challenge recognized the strain-specific HVR, bound selectively to live M75 *S. pyogenes*, and mediated opsonophagocytic killing. These findings are consistent with our earlier observations in preclinical models, which demonstrated long-lasting homologous immunity following a single *S. pyogenes* upper respiratory tract^34^ or skin/superficial^17^ infection. In contrast, Neergaard et al. reported the presence of cross-opsonic antibodies following invasive *S. pyogenes* infection^35^, suggesting that opsonic immunity in this context may not be strictly type-specific. This raises the possibility that superficial and invasive infections differentially shape the breadth of the ensuing antibody response. The anti-M75 IgG response observed in the present study is predominantly mediated by the IgG1 and IgG3 subclasses, which are particularly effective in pathogen clearance, presenting a number of antigen-specific effector functions, including interaction of antigen-clustered IgG with the complement system, which activates phagocytosis and production of reactive oxygen species by innate immune cells^36–38^. Despite its relatively low abundance in relation to IgG1, IgG3 has been increasingly recognized as a key player in the immune defence against various pathogens, including *S. pyogenes*^39^ due to its superior affinity to Fc-gamma receptors^40^. However, excessive IgG3 recognizing M-protein antigens may be detrimental and elevated levels have been linked to the *S. pyogenes* post-infectious syndrome, acute rheumatic fever^41,42^.

Although total and subclass-specific M75-IgG antibody levels prior to challenge did not distinguish between clinical outcomes following experimental challenge, significant differences in IgG Fc N-glycosylation traits both in total and M75-specific IgG1 were observed. The glycosylation features of the Fc region significantly influence the ability of IgG antibodies to trigger inflammatory responses and their interaction with FcγIII receptors^43,44^. Compared to those with symptomatic pharyngitis, asymptomatic participants had higher total IgG1 sialylation, suggesting that the anti-inflammatory properties of Fc sialylation^45,46^ may also contribute to protection. Additionally, they exhibited an absence of afucosylated N-glycans in M75-specific IgG1 Fc, which has been associated with exacerbated inflammatory responses and disease severity following viral infections^47,48^. Our data provide evidence that subclinical exposure to *S. pyogenes* can selectively shape the N-glycan landscape of M75-specific IgG1 antibodies. The concurrent increase in both agalactosylation and sialylation following challenge suggests a fine-tuned immune adaptation of M75-specific responses: the host may be enhancing IgG1 functionality for bacterial clearance (via agalactosylation^49^) while simultaneously limiting collateral inflammation and tissue damage (via sialylation and fucosylation^46,50^). This dual modulation could reflect an evolved mechanism to balance pathogen elimination with immune regulation during asymptomatic *S. pyogenes* exposures. Although the anti-M75 IgG response was predominantly mediated by the IgG3 subclass post-challenge (as per ELISA data), the IgG Fc N-glycosylation in the non-pharyngitis (asymptomatic) participants appeared to be more prominent on IgG1 as compared to the pharyngitis group (both total and M75-IgG). Nevertheless, significant glycosylation changes were only observed in M75-specific IgG1 and not IgG3/4. Importantly, the glycosylation differences observed in asymptomatic participants do not imply increased antibody potency relative to symptomatic participants but rather reflect qualitative differences in Fc profiles that may influence immune regulation and thus infection outcome. It is possible that in asymptomatic participants, Fc remodeling enhances interactions with Fcγ receptors and complement, thereby favoring efficient opsonisation and clearance of bacteria at mucosal surfaces by neutrophils and macrophages. In contrast, symptomatic infection may induce antibodies with stronger inflammatory or complement-activating properties, which could be more effective at controlling bacterial burden within deeper respiratory tissues. However, in the absence of detailed mechanistic data, the underlying mechanisms remain unclear.

The modest immune responses to the conserved C-terminal M-protein peptide p*17 observed in this study are consistent with previous findings in both mice and children from streptococcal-endemic regions^16^. Although C-terminal antigens appear to be cryptic during exposure and infection with *S. pyogenes*, vaccination with the p*17 antigen conjugated to a carrier protein elicits robust protective immune responses in animal models. These include high-affinity, class-switched antibodies that recognize a broad range of *S. pyogenes* strains, effectively reducing bacterial colonization and preventing severe infection^23–26^. These observations underscore the likelihood that vaccine-induced protection against *S. pyogenes* operates through mechanisms distinct from naturally acquired immunity. This is evidenced by continued low susceptibility to infection among adults, particularly in parents of young children, individuals in crowded settings (e.g. army barracks), people who inject drugs, those experiencing homelessness, and older adults with accumulating comorbidities.

A limitation of our study is the small cohort size, especially the limited number of asymptomatic participants. The high rate of clinical pharyngitis in the challenged adult participants, despite pre-existing immunity to antigens other than the type-specific M75-HVR (an exclusion criterion)^21,51^, may suggest the inoculum (dose) and direct swab challenge method may have circumvented or overwhelmed natural defences^21^. Still, the inoculum was several orders of magnitude lower than in comparable to animal models, and around a third of challenged participants remained asymptomatic, and those participants did develop de novo type-specific antibodies. Beyond these model-related concerns, this study was unable to directly manipulate Fc glycosylation in the IgG used for functional studies due to the limited availability of M75-specific antibodies. Future research employing glycoengineered antibodies will be required to clarify the functional role of Fc glycosylation in anti-*S. pyogenes* immunity. Furthermore, as antibody repertoire analyses were not performed, we cannot exclude the possibility that differences in clinical outcomes were also influenced by the generation of distinct antibody clones following challenge. Besides, this study did not consider responses to conserved non-M-protein antigens such as SpyCEP, SLO and ScpA, which have previously been found in this cohort to have been increased in participants with pharyngitis and to have persisted for at least 3 months post-challenge^51^. Following challenge, the increase in serum IgG binding to the M75 strain, but not the heterologous strains, suggests that the responses were dominant against the M-protein but not to other shared surface proteins/antigens. These data suggest a link between Fc N-glycosylation changes and IgG effector activity in *S. pyogenes* infection, but larger studies with M75-specific IgG are needed to clarify its roles in mechanisms such as complement activation and FcγR-mediated phagocytosis.

In conclusion, our study demonstrated that asymptomatic *S. pyogenes* infection can induce persistent serotype-specific functional antibodies. This finding could be re-examined in longitudinal *S. pyogenes* surveillance studies, including serial serum sampling and tested in future human studies incorporating challenge and homologous rechallenge. We propose that subclinical exposures do contribute to the development of protective immunity against *S. pyogenes* in humans.

## Methods

### The CHIVAS-M75 study

All human research conducted in this study complied with relevant ethical regulations and was approved by the Alfred Hospital Human Research Ethics Committee (reference number 500/17). All participants provided written informed consent prior to enrollment. The CHIVAS-M75 study protocol, including information on the M75 *S. pyogenes* challenge strain and key findings, has been detailed in earlier publications^20–22^. Participants were recruited based on voluntary participation and eligibility screening according to predefined inclusion and exclusion criteria outlined in the clinical study protocol^21^. Among the 25 participants enrolled in the trial, 19 developed pharyngitis, across two dose levels. The diagnosis of pharyngitis was corroborated by microbiological evidence (via qPCR and culture), elevated biochemical markers (C-reactive protein, cytokines, and chemokines), and serological responses (anti-streptolysin O and anti-DNase B antibodies). Importantly, all participants classified as asymptomatic in the present study showed no signs of *S. pyogenes* colonization after 24 h. In the present study, analysis was limited by sample availability. Participant sex was determined by self-report. Participants were recruited to ensure representation of both sexes. No difference in the prevalence of pharyngitis was observed between males and females in this cohort. Given the limited sample size (*n* = 18), we did not pursue further sex‑stratified analyses. Serum and PBMC were collected from participants at the evening prior to the challenge, then at 1 month and 6 month outpatient visits. Blood was collected in serum separator tubes (BD Vacutainer SST Gold 8.5 mL) and allowed to clot. Within 2 h of collection, tubes were centrifuged at 1500 × *g* for 15 min at 20 °C, and then aliquots were stored at −80 °C. PBMCs were collected using CPT tubes (BD Bioscience).

### Murine model of *S. pyogenes* infection

All animal protocols were reviewed and approved by the Griffith University Animal Ethics Committee (GU-AEC) in accordance with the National Health and Medical Research Council (NHMRC) of Australia guidelines. Experimental protocols involving SCID (Severe Combined Immunodeficiency) mice were reviewed and approved by Office of the Gene Technology Regulator (OGTR). Female C.B−17/IcrHanHsd-Prkdc^scid^/Ozarc (SCID) mice (Mus musculus) aged 4–6 weeks were sourced from Ozgene, Western Australia. All mice were housed in a PC2-certified animal facility in individually ventilated cages (IVC) with a maximum of 5 mice/cage. For ELISA and binding assays (Supplementary Fig. 1), the mice received three intramuscular injections with p*17-DT/Alum or M75-DT/Alum. For positive controls in the pre-opsonization assay, Sprague Dawley rats aged 9 weeks at study initiation were sourced from Charles River Laboratories, Inc. and immunized with p*17-K4S2-CRM/Alum. In both studies, animals received three intramuscular immunizations over a six-week period, followed by a two-week recovery phase prior to sample collection. The adjuvant, aluminium hydroxide (Alhydrogel 2%, Alum), was obtained from Brenntag Biosector, Denmark. The relative humidity ranged between 45 and 65% and temperature at 20–24 °C. Mice and rats were exposed to a 12-h light–dark cycle.

### Antigens

Synthetic peptides from the M75 protein hypervariable region (M75-HVR) and C-terminal region (p*17) regions were synthesized and purified (>95%) commercially by GenScript and stored lyophilized or in solution at −20 °C. Peptide sequence for M75-HVR is: EEERTFTELPYEARYKAWKSENDELRENYRRTLDKFNTEQGKTTRLEEQN. Peptide sequence for p*17 is: LRRDLDASREAKNQVERALE.

### Enzyme-linked immunosorbent assays (ELISA)

Standard ELISA was used to measure M75 and p*17-specific serum antibody levels. Briefly, peptides were coated onto NUNC MaxiSorp plates (Thermo Fisher Scientific). Antigens were resuspended to 5 µg/mL in 0.1 M coating carbonate buffer and coated at 100 µL per well onto 96-well medium-binding ELISA plates (Greiner Bio-One) overnight at room temperature. Plates were then blocked with 200 µL of 2% w/v bovine serum albumin (BSA, Sigma-Aldrich) in phosphate-buffered saline (PBS) overnight at 4 °C, followed by five washes in wash buffer (PBS with 0.05% v/v Tween20, PBS-T). Serial dilutions were performed using diluent (0.05% v/v Tween20) at 1:10, 1:20, 1:40, 1:80 dilutions. Samples were diluted 2-fold and antibody levels detected with HRP-conjugated antibodies. Anti-human antibodies included IgA (#A18781, polyclonal), IgG (H + L) (#31135, polyclonal), IgG1 Fc (#A−10648, clone HP6069), IgG2 (#MH1722, clone HP6014), IgG3 hinge (#05-3620, clone HP6047) and IgG4 Fc (#A−10654, clone HP6025) (Invitrogen/Thermo Fisher Scientific). Mouse ELISA experiments used goat anti-mouse IgG (H + L)-HRP (#1706516, polyclonal). SIGMAFAST OPD substrate was added according to manufacturer’s instructions and absorbance was measured at 450 nm on a Tecan Infinite M200 Pro plate reader (Tecan Group Ltd.). Negative controls were included in each plate containing commercial human sera (Sigma-Aldrich) pre-absorbed of anti-M75 *S. pyogenes* antibodies or containing 0.05% v/v Tween20 only.

### Tetramer staining for peptide-specific memory B cells

Frozen mononuclear PBMCs were thawed at 37 °C in thawing media (RPMI + 10% FCS), then washed and resuspended in flow buffer (PBS + 2%FCS + 2 mM EDTA). Biotinylated peptides synthesized at Genscript were tetramerised by serial addition of premium-grade streptavidin-PE and streptavidin-APC (BioLegend) at a four-molar equivalent of biotinylated peptide to one molar equivalent of streptavidin during 2 h at room temperature^52^. The prepared tetramer was diluted to a concentration of 2 μm for use. Typically, 10^6^ PBMCs were pre-incubated with Fc block (BioLegend) for 10 min followed by tetramer staining for 40 min. Cells were washed with flow buffer and stained with fluorochrome-conjugated antibodies: CD19 BUV395 (#302239, clone HIB19, 1:200), CD21 FITC (#354910, clone Bu32, 1:200), CD27 BV421 (#356417, clone M-T271, 1:400), IgD PE/Dazzle 594 (#348239, clone IA6-2, 1:400), IgM BV711 (#314539, clone MHM-88, 1:200), IgG Fc BV510 (#410716, clone M1310G05, 1:200) (BioLegend), and IgA PerCP Vio700 (#130−113-478, clone IS11-8E10, 1:300; Miltenyi Biotec).Cells were fixed with 1% PFA, acquired on a LSRFortessa (BD Biosciences) and analyzed using FlowJo™ v11 Software (BD Biosciences). M75- and p*17-specific cells were gated as positive for both fluorochromes of an antigen tetramer pair to reduce the inclusion of non-specific binding cells, as shown in Fig. 1 and 2.

### *S. pyogenes* strains and growth conditions

A number of *S. pyogenes* isolates sourced from various laboratories were utilized in this study. These included 5448 (emm1), isolated from a case of invasive infection^53^ (provided by Prof. Mark Walker, University of QLD, BNE, Australia), M12 (Menzies School of Health Research, Darwin, Australia), M59 (Prof. Greg Tyrrell, University of Alberta, Canada), and M89 (Prof. Jenny Robson, Sullivan & Nicolaides Pathology, BNE, Australia). *S. pyogenes* strain 611024 (emm75, GenBank accession CP033621), isolated in Melbourne from a child presenting with exudative pharyngitis, was used in this study^21^. For mouse infection experiments, M75 *S. pyogenes* was rendered streptomycin-resistant (200 μg/mL) to enable selective recovery from commensal flora in the respiratory mucosa and tissues.

Single colonies were inoculated into Todd Hewitt Broth (THB; Oxoid, Australia) supplemented with 1% Bacto Neopeptone (Thermo Fisher Scientific), yeast extract and streptomycin^24^. To enumerate colony-forming units (CFU), cultures were plated on Columbia agar containing 5% defibrinated horse blood and 200 μg/ml streptomycin (CBA 5%)^24^.

### IgG purification

IgG antibodies were purified from the serum of CHIVAS participants and immunized rats using NAb Protein G columns, following the manufacturer’s instructions (Thermo Fisher, USA). The concentration of purified IgG was measured using a NanoDrop spectrophotometer (Thermo Fisher, USA).

M75 antigen-specific IgG antibodies were purified using SulfoLink™ affinity chromatography columns (Thermo Fisher Scientific) following the manufacturer’s protocol. Briefly, the columns were prepared by coupling M75-HVR to the SulfoLink resin via sulfhydryl-reactive chemistry. Serum samples were then applied to the columns, allowing M75-HVR-specific IgG to bind selectively. After thorough washing to remove unbound proteins, antigen-specific IgG was eluted using a low pH glycine buffer and immediately neutralized. The purified IgG fractions were concentrated and buffer-exchanged into PBS using centrifugal filters (Amicon Ultra, Millipore). Purity of the eluted M75-specific IgG was assessed by silver protein stain (Thermo Fisher Scientific) and ELISA. Purified antibodies were stored at –20 °C until further use. A negative control was generated by incubating human IgG with multiple *S. pyogenes* isolates, including those relevant to this study (M1 and M75). Following overnight incubation, the bacterial pellets were removed by centrifugation, and the adsorbed IgG preparation was collected.

### IgG binding to *S. pyogenes*

For the binding assay, M1 and M75 *S. pyogenes* strains (0.5 O.D.) were incubated overnight at 4 °C in PBS containing 0.2% skim milk PBS with 100 µg of purified serum IgG collected from immunized mice or CHIVAS participants at pre-challenge and 6 months post-challenge. Following incubation, bacterial cells were washed and stained with anti-human IgG Fab secondary antibody PE (#MA1−10377, clone 4A11, 1:200; Invitrogen) and anti-mouse IgG1 PE/Dazzle 594 (#406628, clone RMG1−1, 1:200; BioLegend). Cells were fixed with 1% PFA, acquired on a LSRFortessa flow cytometer (BD Biosciences) and analyzed using FlowJo™ v11 Software (BD Biosciences).

### Pre-opsonization of *S. pyogenes* with IgG and intranasal challenge in mice

M75 *S. pyogenes* was incubated with purified serum IgG collected from CHIVAS participants at pre-challenge and 6 months post-challenge at room temperature for 45 min. As controls, IgG was purified from Sprague Dawley rats either immunized with p*17-DT-K4S2-CRM/Alum or left unimmunized (naïve). 10 µL of IgG (30 mg/mL) was incubated with 5 µL of stationary phase live bacteria (OD600 = 0.5) for 45 minutes at room temperature. Following incubation, 5 × 10^7^ CFU/mouse was delivered intranasally to anaesthetized naive SCID mice. The protocol was a modification of a previous assay^24^. Throat swabs were collected using flocked swabs (Copan Diagnostics), which were then suspended in PBS, serially diluted, and plated in duplicate on CBA 5% plates. To assess nasal shedding, each mouse had its nares gently pressed onto a CBA 5% plates ten times (five times per half plate), allowing expelled particles to be streaked across the surface. On day 2 post-infection, mice were euthanized using CO₂ asphyxiation. The nasal-associated lymphoid tissue (NALT), a murine analogue to human tonsils, and the lungs were collected, homogenized in PBS using a Bullet Blender Homogenizer (Next Advance), and serially diluted before being plated onto CBA 5% plates. Mice were observed daily for clinical signs of illness according to a monitoring sheet approved by the GU-AEC^24^. Clinical parameters were scored on a scale of 1–3 for the following criteria: ruffled fur, hunched posture, responsiveness to stimuli, level of consciousness/activity, respiratory quality, ocular secretions, presence of blood in throat swabs, and body weight loss relative to pre-challenge measurements. Mice were humanely euthanized immediately if a score greater than 3 was recorded for any individual parameter or if the cumulative clinical score exceeded 14.

### IgG glycopeptide preparation

Total IgG levels were quantified using two technical replicates per individual. For M75 antigen‑specific IgG, one biological sample per participant was analyzed (*n* = 5), and each sample was measured in technical duplicate. Samples were vacuum‑dried and reconstituted in 25 µL of 25 mM ammonium bicarbonate, followed by tryptic digestion at an enzyme‑to‑substrate ratio of 1:25 at 37 °C for 3 h, as previously described ^54^. The resulting trypsin-digested glycopeptides were dried and redissolved in 0.1% trifluoroacetic acid prior to desalting using porous graphitized carbon (PGC). Approximately 25 µL of PGC resin was packed onto a 10 µL C18 ZipTip and conditioned with 80% acetonitrile in 0.1% trifluoracetic acid (three washes), followed by equilibration with 0.1% trifluoroacetic acid (three washes). Reconstituted peptide eluates (50 µL, 0.1% trifluoroacetic acid) were loaded, washed three times, and (glyco)peptides were eluted twice using 50% acetonitrile in 0.1% trifluoracetic acid. Desalted glycopeptides were dried and redissolved in 0.1% trifluoracetic acid prior to LC-MS analysis.

### IgG Fc-glycopeptide profiling by LC-ESI MS/MS

IgG glycopeptides were analyzed using an Orbitrap Fusion mass spectrometer (Thermo Fisher Scientific), fitted with a PicoTip nanospray interface (New Objective), trap column (pre-column) and analytical reversed-phase C18 PepMap Neo (75 μm × 500 mm) UHPLC column (Thermo Fisher Scientific). IgG glycopeptides, including glycopeptides derived from a commercial IgG standard were injected onto the pre-column in 100% solvent A (Loading Buffer: 0.1% trifluoracetic acid) at the flow rate of 15 µL/min and the glycopeptides were separated at a flow rate of 300 nL/min using solvent B (NC Buffer: 80% acetonitrile with 0.1% formic acid). A 40-min linear gradient (8 to 95%) was applied as follows: 1% solvent B from 0 to 8 min, 8% from 8 to 9 min, 8–15% from 9 to 20 min, 15–95% from 20 to 23 min, 95% from 23 to 25 min, and 1% solvent B from 28 to 40 min. All spectra were acquired in positive ion mode in the data-dependent (DDA) mode. For MS acquisition, a full MS1 scan was acquired at a resolution of 60,000 over an *m/z* range of 350–1650 (custom AGC target of 250%, maximum injection time of 50 ms, one microscan). This was followed by Parallel Reaction Monitoring (PRM) at 60,000 resolution, triggered using a scheduled inclusion list comprising predefined IgG1, IgG2, IgG3, and IgG4 glycopeptide precursor masses and retention times. Fragmentation spectra were obtained with an isolation window of 1.6 Da using normalized stepped-HCD collision energy (15, 30, 45%).

For intact IgG glycopeptide identification, the LC-MS/MS raw data files (.raw) were searched using Byonic^TM^ version 5.4.10 (Protein Metrics Inc) against the UniProt *Homo sapiens* database (12 September 2019 release; 74,468 entries) with corresponding *N*-glycoforms searched using the in-built Byonic library containing 309 human *N*-glycan entries. The trypsin enzymatic cleavage at the C-terminal side of the amino acid residues lysine (K) and arginine (R) was selected with a maximum of two missed cleavages allowed. For HCD MS/MS setting, the precursor mass tolerance was set to 3 ppm, while fragment mass tolerance was set at 10 ppm. All data was searched with peptide spectrum matches (PSM) FDR set to 1% with a minimum Byonic score of 200 to ensure high confidence identifications. Fixed modifications comprised of carbamidomethylation of cysteine residues ( + 57.0215 Da) and dynamic modifications consisted of oxidation of methionine ( + 15.9949 Da), acetylation ( + 42.011 Da) and deamidation ( + 0.984 Da) modifications at asparagine residues.

Raw LC–MS data were processed using established in-house glycoproteomics identification workflows, with reference to pre-defined glycopeptide masses derived from a commercial IgG standard. Glycopeptide annotations were assigned based on accurate mass, retention time, and MS/MS fragmentation patterns, and were validated by diagnostic fragment ions, including IgG subclass-specific Y1 ions. Raw data (.raw) were imported into Skyline (version 25.1.0.142; MacCoss Lab, University of Washington) for targeted extraction and quantitative analysis^55^. Transition settings were configured for full-scan filtering of precursor and fragment ions. For MS1 filtering, full-scan precursor extraction was enabled using isotope-percent filtering, retaining isotopes with a minimum intensity of 20% of the base peak. MS1 spectra were extracted at a resolving power of 60,000 at *m/z* 400, including precursor charge states 1+ to 3 + . For MS/MS filtering, full-scan fragment ion extraction was enabled using Orbitrap MS/MS spectra acquired in data-dependent acquisition (DDA) mode. Fragment ions were extracted with a mass accuracy tolerance of 20 ppm, applying a resolving power of 60,000 at *m/z* 400. Retention time filtering was applied using a ± 5 min window centered on the identified glycopeptide elution time. The mass range for extraction was set from *m/z* 50 to *m/z* 1800, with a method match tolerance of *m/z* 0.05.

All quantitative data processing was performed using Skyline based on the following settings: MS1 peak-area-under-the curve quantification was performed based on IgG glycopeptide masses corresponding to doubly [M + 2H]^2+^ and triply [M + 3H]^3+^ charged precursor masses (Supplementary Table 1) and the summed areas of integrated peaks (including monoisotopic transitions) for each glycopeptide precursor mass were represented as a percentage (relative intensity) of the total IgG glycopeptides. For samples analyzed with duplicate technical injections, intensities were averaged prior to downstream analysis.

### Statistical analysis

Statistical analysis was performed with GraphPad Prism 9 software using one-way ANOVA with Tukey’s post-hoc test was used for multiple paired comparisons, whereas a two-tailed paired *t*-test was applied when comparing only two groups pre and post-challenge. Effect sizes with 95% CIs alongside *p*-values are included. Unpaired *t*-test was used for statistical analysis when comparing between participants with and without pharyngitis and for comparing pre versus post. ARRIVE guidelines^56^ were used to calculate sample size for in vivo mouse experiments. A sample size of 8 was shown to provide a power of 0.8 (G*Power) and therefore used for animal experiments involving bacterial challenge. Data were excluded based on an outlier test performed in GraphPad Prism using the ROUT (Robust regression and Outlier Removal) method, which applies a nonlinear regression model and a 2% false discovery rate to identify outliers.

For ELISA analysis, absorbance values at a 1:20 serum dilution were used to compare antigen-specific responses across the three time points. Correlation coefficients and correlation *p* values were determined using Spearman’s method (two-sided) with a linear regression line included to indicate linear trends.

### Reporting summary

Further information on research design is available in the Nature Portfolio Reporting Summary linked to this article.

## Supplementary information

Supplementary Information

Reporting Summary

Transparent Peer Review file

## Source data

Source Data 1

Source Data 2

## Data Availability

All raw mass spectrometry data generated in this study and associated metadata have been deposited in the GlycoPOST repository^57^ and are publicly accessible under the accession number (10.50821/GLYCOPOST-GPST000603). Source data are provided with this paper.
