# Supplementary material for: Subclinical exposure to Streptococcus pyogenes drives the development of long-lived homologous immunity
Source: Nat Commun. 2026 Aug 12;17:7850. doi: 10.1038/s41467-026-76176-1 (PMC13470482; doi:10.1038/s41467-026-76176-1)
Supplement: Supplementary file 1 [file 41467_2026_76176_MOESM1_ESM.pdf]

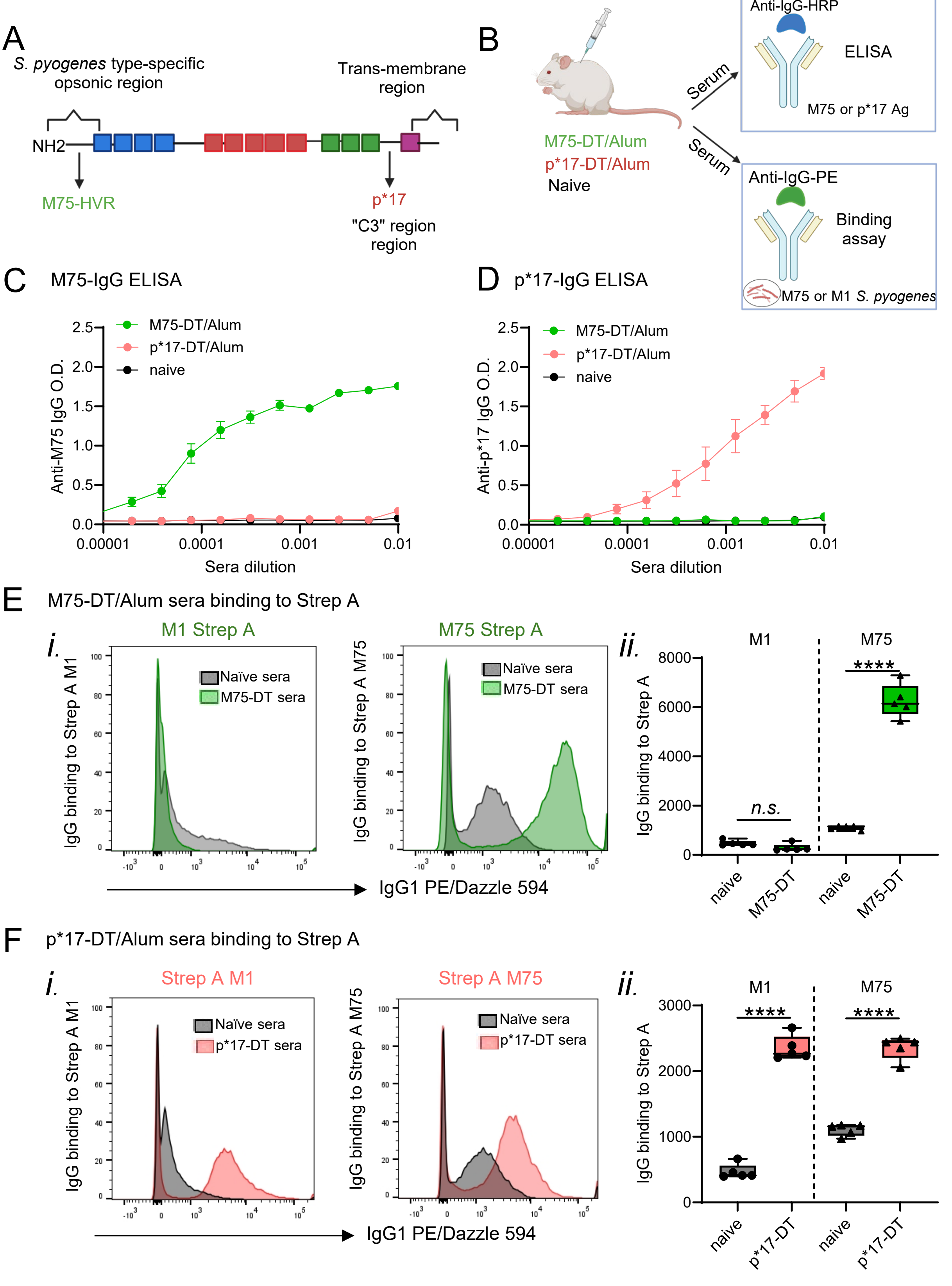

A
S. pyogenes type-specific opsonic region
Trans-membrane region
NH2
M75-HVR
p*17
"C3" region region
B
M75-DT/Alum
p*17-DT/Alum
Naive
Serum
Serum
Anti-IgG-HRP
ELISA
M75 or p*17 Ag
Anti-IgG-PE
Binding assay
M75 or M1 S. pyogenes
C
M75-IgG ELISA
M75-DT/Alum
p*17-DT/Alum
naive
Anti-M75 IgG O.D.
Sera dilution
D
p*17-IgG ELISA
M75-DT/Alum
p*17-DT/Alum
naive
Anti-p*17 IgG O.D.
Sera dilution
E
M75-DT/Alum sera binding to Strep A
i.
M1 Strep A
M75 Strep A
Naïve sera
M75-DT sera
IgG binding to Strep A M1
IgG binding to Strep A M75
IgG1 PE/Dazzle 594
ii.
M1
M75
****
n.s.
IgG binding to Strep A
naive
M75-DT
naive
M75-DT
F
p*17-DT/Alum sera binding to Strep A
i.
Strep A M1
Strep A M75
Naïve sera
p*17-DT sera
IgG binding to Strep A M1
IgG binding to Strep A M75
IgG1 PE/Dazzle 594
ii.
M1
M75
****
****
IgG binding to Strep A
naive
p*17-DT
naive
p*17-DT

Supplementary Figure 1. Domain localization, antibody responses, and *S. pyogenes* binding following immunization with M75-HVR and p*17 epitope-based vaccines. Created in BioRender. Pandey, M. (2026) https://BioRender.com/iwnco2k. (A) Schematic representation of M-protein domains indicating the N-terminal position of M75 epitope, which is derived from the HVR region of the M-protein (M75-HVR), and the highly conserved p*17 epitope, derived from the C-terminal (C3) position. (B) Sera collected two weeks after the final immunization of BALB/c mice with p*17-DT or M75-DT adjuvanted with Alum (n=5/group) were assessed using direct ELISA and S. pyogenes binding assays. (C and D) ELISA was used to detect (C) anti-M75 and (D) anti-p*17-specific IgG at O.D. 450 nm. Absorbance values are shown for sera collected from mice immunized with M75-DT/Alum, p*17-DT/Alum or naïve controls at multiple dilutions. (E and F) Flow cytometry-based assay analyzing the binding of sera collected from mice immunized with (E) M75-DT/Alum or (F) p*17-DT/Alum to M1 and M75 live *S. pyogenes*. Serum obtained from naïve mice was used as controls. i. Representative histograms illustrating S. pyogenes binding by sera from one mouse per group. ii. Box plots depicting IgG1 antibody binding to *S. pyogenes*, quantified as mean fluorescence intensity (MFI). Unpaired t-tests were used for statistical analysis. n.s. non-significant; ****P<0.0001. Source data are provided as a Source Data file.

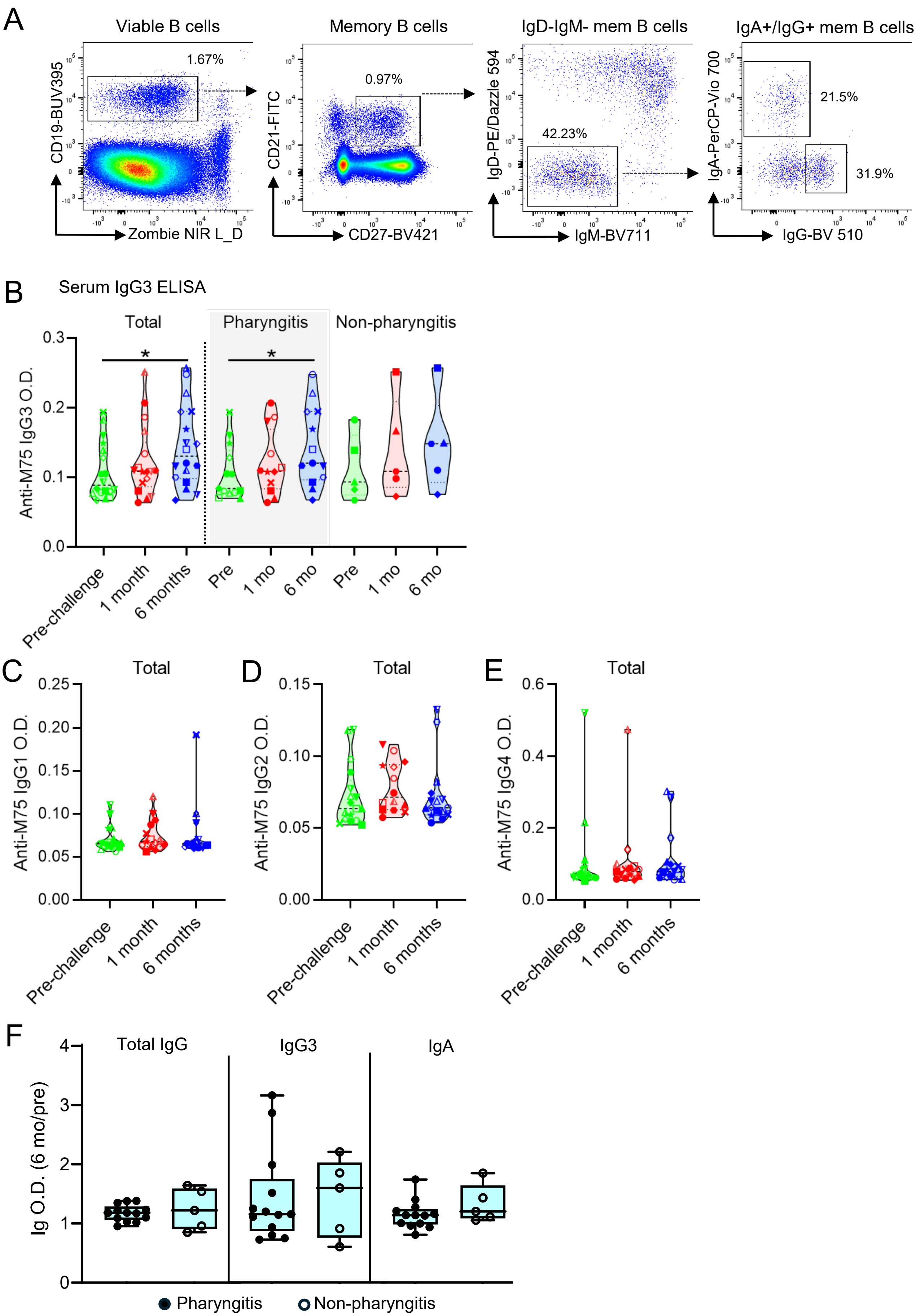
A
Viable B cells
1.67%
CD19-BUV395
Zombie NIR L_D
Memory B cells
0.97%
CD21-FITC
CD27-BV421
IgD-IgM- mem B cells
42.23%
IgD-PE/Dazzle 594
IgM-BV711
IgA+/IgG+ mem B cells
21.5%
31.9%
IgA-PerCP-Vio 700
IgG-BV 510
B
Serum IgG3 ELISA
Total
Pharyngitis
Non-pharyngitis
Anti-M75 IgG3 O.D.
Pre-challenge
1 month
6 months
Pre
1 mo
6 mo
C
Total
Anti-M75 IgG1 O.D.
D
Total
Anti-M75 IgG2 O.D.
E
Total
Anti-M75 IgG4 O.D.
F
Total IgG
IgG3
IgA
Ig O.D. (6 mo/pre)
Pharyngitis
Non-pharyngitis

Supplementary Figure 2. Antigen-specific immune responses to M75-HVR following experimental human Strep A M75 infection. (A) Flow cytometry gating strategy for analysis of IgG^{+} and IgA^{+} memory B cells in the peripheral blood. (B,C)) ELISA was used to detect anti-M75-specific IgG antibody subtypes in serum samples. Violin plots show O.D. 450 absorbance values are shown for sera collected at pre-challenge (day -1), 1 month post-challenge and 6 months post-challenge, using a 1:20 dilution. Violine plots show the absorbance values of M75-specific (IgG3 (C) IgG1, (D) IgG2 and (E) IgG4. Violin plots show O.D. 450 absorbance values for sera collected at pre-challenge (day -1) and 6 months post-challenge, using a 1:20 dilution. Data are stratified by participants who developed pharyngitis and those who did not. Each individual is represented by a symbol. One-way ANOVA followed by Bonferroni's comparisons tests were performed in all statistical analyses. *P<0.05, **P<0.01. (F) Box and whiskers plot of the ELISA data showing OD fold-change (6 months/ pre-infection) for M75-specific total IgG, IgG3, and IgA at a 1:20 dilution. Median and 25th, and 75th percentiles (boxes), as well as the minimum and maximum values, are indicated. The fold change represents the difference in OD values of 6 months post-infection compared to pre-infection levels. Source data are provided as a Source Data file.

Retention Time	Glycan ID	Glycan Structure	Precursor Mass	
			[M+3H]$^{3+}$	[M+2H]$^{2+}$
**IgG1**				
23.5-24.5	IgG1_G0F	Hex[3]HexNAc[4]Fuc[1]	878.6868	1317.5266
23.5-24.5	IgG1_G1F	Hex[4]HexNAc[4]Fuc[1]	932.7044	1398.5530
23.5-24.5	IgG1_G2F	Hex[5]HexNAc[4]Fuc[1]	986.7220	1479.5794
23.5-24.5	IgG1_G0	Hex[3]HexNAc[4]	830.0008	1244.4976
23.5-24.5	IgG1_G1	Hex[4]HexNAc[4]	884.0184	1325.5240
23.5-24.5	IgG1_G2	Hex[5]HexNAc[4]	938.0361	1406.5504
23.5-24.5	IgG1_G0B	Hex[3]HexNAc[5]	897.6940	1346.0373
23.5-24.5	IgG1_G0FB	Hex[3]HexNAc[5]Fuc[1]	946.3799	1419.0663
23.5-24.5	IgG1_G1FB	Hex[4]HexNAc[5]Fuc[1]	1000.3975	1500.0927
23.5-24.5	IgG1_G2FB	Hex[5]HexNAc[5]Fuc[1]	1054.4151	1581.1191
25.4-26.0	IgG1_G1FS	Hex[4]HexNAc[4]Fuc[1]NeuAc	1029.7362	1544.1007
25.4-26.0	IgG1_G2FS	Hex[5]HexNAc[4]Fuc[1]NeuAc	1083.7538	1625.1271
25.4-26.0	IgG1_G2FS2	Hex[5]HexNAc[4]Fuc[1]NeuAc[2]	1180.7856	1770.6748
25.4-26.0	IgG1_G1FBS	Hex[4]HexNAc[5]Fuc[1]NeuAc	1097.4293	1645.6404
25.4-26.0	IgG1_G2FBS	Hex[5]HexNAc[5]Fuc[1]NeuAc	1151.4470	1726.6668
23.5-24.5	IgG1_G1B	Hex[4]HexNAc[5]	951.7116	1427.0637
23.5-24.5	IgG1_G2B	Hex[5]HexNAc[5]	1005.7292	1508.0901
25.4-26.0	IgG1_G1S	Hex[4]HexNAc[4]NeuAc	981.0503	1471.0717
25.4-26.0	IgG1_G2S	Hex[5]HexNAc[4]NeuAc	1035.0679	1552.0982
**IgG2**				
28.0-29.5	IgG2_G0F	Hex[3]HexNAc[4]Fuc[1]	868.0235	1301.5317
28.0-29.5	IgG2_G1F	Hex[4]HexNAc[4]Fuc[1]	922.0411	1382.5581
28.0-29.5	IgG2_G2F	Hex[5]HexNAc[4]Fuc[1]	976.0587	1463.5845
28.0-29.5	IgG2_G0	Hex[3]HexNAc[4]	819.3376	1228.5027
28.0-29.5	IgG2_G1	Hex[4]HexNAc[4]	873.3552	1309.5291
28.0-29.5	IgG2_G2	Hex[5]HexNAc[4]	927.3728	1390.5555
28.0-29.5	IgG2_G0B	Hex[3]HexNAc[5]	887.0307	1330.0424
28.0-29.5	IgG2_G0FB	Hex[3]HexNAc[5]Fuc[1]	935.7167	1403.0713
28.0-29.5	IgG2_G1FB	Hex[4]HexNAc[5]Fuc[1]	989.7343	1484.0978
28.0-29.5	IgG2_G2FB	Hex[5]HexNAc[5]Fuc[1]	1043.7519	1565.1242
29.5-31	IgG2_G1FS	Hex[4]HexNAc[4]Fuc[1]NeuAc	1019.0729	1528.1058
29.5-31	IgG2_G2FS	Hex[5]HexNAc[4]Fuc[1]NeuAc	1073.0906	1609.1322
29.5-31	IgG2_G2FS2	Hex[5]HexNAc[4]Fuc[1]NeuAc[2]	1170.1224	1754.6799
29.5-31	IgG2_G1FBS	Hex[4]HexNAc[5]Fuc[1]NeuAc	1086.7661	1629.6455
29.5-31	IgG2_G2FBS	Hex[5]HexNAc[5]Fuc[1]NeuAc	1140.7837	1710.6719
28.0-29.5	IgG2_G1B	Hex[4]HexNAc[5]	941.0483	1411.0688
28.0-29.5	IgG2_G2B	Hex[5]HexNAc[5]	995.0659	1492.0952
29.5-31	IgG2_G1S	Hex[4]HexNAc[4]NeuAc	970.3781	1455.0768
29.5-31	IgG2_G2S	Hex[5]HexNAc[4]NeuAc	1024.4046	1536.1032
**IgG3/4**				
26.2-27	IgG3/4_G0F	Hex[3]HexNAc[4]Fuc[1]	873.3552	1309.5291
26.2-27	IgG3/4_G1F	Hex[4]HexNAc[4]Fuc[1]	927.3728	1390.5555
26.2-27	IgG3/4_G2F	Hex[5]HexNAc[4]Fuc[1]	981.3904	1471.5819
26.2-27	IgG3/4_G0	Hex[3]HexNAc[4]	824.6692	1236.5002
26.2-27	IgG3/4_G1	Hex[4]HexNAc[4]	878.6868	1317.5266
26.2-27	IgG3/4_G2	Hex[5]HexNAc[4]	932.7044	1398.553
26.2-27	IgG3/4_G0B	Hex[3]HexNAc[5]	892.3623	1338.0399
26.2-27	IgG3/4_G0FB	Hex[3]HexNAc[5]Fuc[1]	941.0483	1411.0688

26.2-27	IgG3/4_G1FB	Hex[4]HexNAc[5]Fuc[1]	995.0659	1492.0952
26.2-27	IgG3/4_G2FB	Hex[5]HexNAc[5]Fuc[1]	1049.0835	1573.1216
29-30	IgG3/4_G1FS	Hex[4]HexNAc[4]Fuc[1]NeuAc	1024.4046	1536.1032
29-30	IgG3/4_G2FS	Hex[5]HexNAc[4]Fuc[1]NeuAc	1078.4222	1617.1297
29-30	IgG3/4_G2FS2	Hex[5]HexNAc[4]Fuc[1]NeuAc[2]	1175.454	1762.6774
29-30	IgG3/4_G1FBS	Hex[4]HexNAc[5]Fuc[1]NeuAc	1092.0977	1637.6429
29-30	IgG3/4_G2FBS	Hex[5]HexNAc[5]Fuc[1]NeuAc	1146.1153	1718.6693
26.2-27	IgG3/4_G1B	Hex[4]HexNAc[5]	946.3799	1419.0663
26.2-27	IgG3/4_G2B	Hex[5]HexNAc[5]	1000.3975	1500.0927
29-30	IgG3/4_G1S	Hex[4]HexNAc[4]NeuAc	975.7186	1463.0743
29-30	IgG3/4_G2S	Hex[5]HexNAc[4]NeuAc	1029.7362	1544.1007

Table 1: List of IgG precursor MS1 glycopeptide masses depicted as doubly $[M+2H]^{2+}$ and triply $[M+3H]^{3+}$ charged masses used for Skyline quantification. Retention times used for MS1 precursor selection and integration of area-under-the curve are indicative of retention time shifts observed using C18 column. MS1 precursors selected for quantification were manually verified based on presence of glycopeptide-specific backbone ion [Pep+HexNAc: Y1_IgG1: *m/z* 1392.5914^{1+}; Y1_IgG2: *m/z* 1360.6016^{1+} and Y1_IgG3/4: *m/z* 1376.5965^{1+}) and oxonium ion (*m/z* 204.0869^{1+}).
