# Supplementary material for: Subclinical exposure to Streptococcus pyogenes drives the development of long-lived homologous immunity
Source: Nat Commun. 2026 Aug 12;17:7850. doi: 10.1038/s41467-026-76176-1 (PMC13470482; doi:10.1038/s41467-026-76176-1)
Supplement: Supplementary file 2 [file 41467_2026_76176_MOESM2_ESM.pdf]

nature portfolio

Corresponding author(s): A/Prof MANISHA PANDEY
Prof MICHAEL GOOD
Dr AILIN LEPLETIER

Last updated by author(s): May 25, 2026

Reporting Summary

Nature Portfolio wishes to improve the reproducibility of the work that we publish. This form provides structure for consistency and transparency in reporting. For further information on Nature Portfolio policies, see our Editorial Policies and the Editorial Policy Checklist.

Statistics

For all statistical analyses, confirm that the following items are present in the figure legend, table legend, main text, or Methods section.

n/a	Confirmed	
☐	☒	The exact sample size (n) for each experimental group/condition, given as a discrete number and unit of measurement
☐	☒	A statement on whether measurements were taken from distinct samples or whether the same sample was measured repeatedly
☐	☒	The statistical test(s) used AND whether they are one- or two-sided<br>*Only common tests should be described solely by name; describe more complex techniques in the Methods section.*
☐	☒	A description of all covariates tested
☐	☒	A description of any assumptions or corrections, such as tests of normality and adjustment for multiple comparisons
☐	☒	A full description of the statistical parameters including central tendency (e.g. means) or other basic estimates (e.g. regression coefficient) AND variation (e.g. standard deviation) or associated estimates of uncertainty (e.g. confidence intervals)
☐	☒	For null hypothesis testing, the test statistic (e.g. F, t, r) with confidence intervals, effect sizes, degrees of freedom and P value noted<br>*Give P values as exact values whenever suitable.*
☒	☐	For Bayesian analysis, information on the choice of priors and Markov chain Monte Carlo settings
☒	☐	For hierarchical and complex designs, identification of the appropriate level for tests and full reporting of outcomes
☐	☒	Estimates of effect sizes (e.g. Cohen's d, Pearson's r), indicating how they were calculated

Our web collection on statistics for biologists contains articles on many of the points above.

Software and code

Policy information about availability of computer code

Data collection	LSR Fortessa (BD)- flow cytometry<br>Tecan Infinite M200 Pro plate reader (Tecan Group Ltd) - ELISA<br>Orbitrap Fusion Tribrid mass spectrometer (Thermo Scientific) - IgG Fc-glycopeptide profiling
Data analysis	FlowJo_v10.10 - flow cytometry<br>Skyline (version 24.1) - IgG Fc-glycopeptide profiling<br>For all experiments, difference between groups were determined using GraphPad Prism version 10

For manuscripts utilizing custom algorithms or software that are central to the research but not yet described in published literature, software must be made available to editors and reviewers. We strongly encourage code deposition in a community repository (e.g. GitHub). See the Nature Portfolio guidelines for submitting code & software for further information.

Data

Policy information about availability of data

All manuscripts must include a data availability statement. This statement should provide the following information, where applicable:

- Accession codes, unique identifiers, or web links for publicly available datasets
- A description of any restrictions on data availability
- For clinical datasets or third party data, please ensure that the statement adheres to our policy

The IgG glycopeptide MS-raw data files have been made available via GlycoPost 55. For the reviewing process, the data is just available with the following information:
Preview Page link:: https://glycopost.glycosmos.org/preview/69820049068637b110aa34
ID: GPST000603, PIN CODE: 7330.

Research involving human participants, their data, or biological material

Policy information about studies with human participants or human data. See also policy information about sex, gender (identity/presentation), and sexual orientation and race, ethnicity and racism.

Reporting on sex and gender	Participant sex was determined by self report. Participants were recruited to ensure representation of both sexes. No difference in the prevalence of pharyngitis was observed between males and females in this cohort. Given the limited sample size (n = 18), we did not pursue further sex-stratified analyses, as any observed differences would be more likely to reflect inter-individual variability rather than a true sex-based effect on immune responses.
Reporting on race, ethnicity, or other socially relevant groupings	The CHIVAS-M75 participants included 12 of 18 individuals who identified as White, with the remainder identifying as belonging to various Asian ethnic groups. With respect to sex and gender, there was a general effort to recruit a balanced sample, but without purposeful stratification and with too few total participants to enable meaningful analyses by race or ethnicity.
Population characteristics	18 healthy adults, 10 males and 8 females. Mean age was 27. 2 years old. 13 symptomatic and 5 asymptomatic participants.
Recruitment	Recruitment of participants was originally described in "Osowicki, J et al. A controlled human infection model of Streptococcus pyogenes pharyngitis (CHIVAS-M75): an observational study. The Lancet Microbe, 2021." Participants were recruited as part of a controlled human infection study involving experimental Streptococcus pyogenes (emm75) exposure in healthy adult volunteers. Recruitment was based on voluntary participation and eligibility screening according to predefined inclusion and exclusion criteria outlined in the clinical study protocol. Participants provided informed consent prior to enrolment.
Ethics oversight	Ethical approval for the study, including sample collection and associated immunological investigations, was granted by the Alfred Hospital Human Research Ethics Committee (reference number 500/17), and all participants provided written informed consent.

Note that full information on the approval of the study protocol must also be provided in the manuscript.

Field-specific reporting

Please select the one below that is the best fit for your research. If you are not sure, read the appropriate sections before making your selection.

[x] Life sciences [ ] Behavioural & social sciences [ ] Ecological, evolutionary & environmental sciences

For a reference copy of the document with all sections, see nature.com/documents/nr-reporting-summary-flat.pdf

Life sciences study design

All studies must disclose on these points even when the disclosure is negative.

Sample size	Sample sizes for human studies were primarily determined by participant and sample availability, including PBMC availability and IgG yield following purification from clinical specimens. All available eligible PBMC samples and corresponding serum samples were included in the analyses shown in Figures 1 and 2. For downstream functional assays requiring purified IgG (Figures 3–6), subset analyses were performed due to limited sample availability and insufficient IgG yield to support all assays across the full cohort. In these experiments, five pharyngitis samples were randomly selected to match the five available asymptomatic samples, and additional pharyngitis samples were included in selected binding assays to confirm that findings were representative of the broader cohort.<br>As reported in the manuscript, ARRIVE guidelines 56 were used to calculate sample size for experiments in mice. A sample size of 8 was shown to provide a power of 0.8 (G*Power) and therefore used for animal experiments involving bacterial challenge.
Data exclusions	All datasets were screened for outliers using the ROUT (Robust Regression and Outlier Removal) method in GraphPad Prism (Q = 2%) prior to statistical analysis. exclusion criteria were pre-established and applied consistently across experiments. No samples were excluded from experiments involving human participants (Figs. 1–5), with the exception of one participant who was excluded from the p17 tetramer IgG

	analysis and p17-specific IgA ELISA due to unusually high pre-existing antibody levels that were not representative of the study cohort. Details of sample exclusion and statistical analyses are provided in the Materials and Methods section.
Replication	Flow cytometry tetramer data of PBMCs could not be replicated due to limited s all attempts at replication were successful.ple a ELISA and binding assays were replicated in 2–3 independent experiments. Fc glycosylation of M75-specific IgG was assessed as the average of two technical replicates and could not be replicated due to sample availability. In vivo assays in SCID mice to evaluate the protective efficacy of IgG were conducted in two independent experiments, using the same positive and negative controls in each experiment. All attempts at replication were successful.
Randomization	All available PBMC samples were included in the study, and corresponding serum ELISA data were matched to the PBMC cohort (Figures 1 and 2). Due to limited sample availability and insufficient IgG yield to perform all downstream assays across the entire cohort, five pharyngitis samples were randomly selected for the experiments included in Figures 3–6 to match the number of available asymptomatic samples. All five asymptomatic samples were included. Samples from symptomatic participants used for IgG binding assays, antigen-specific IgG purification and glycosylation analyses, and in vivo transfer experiments were representative of the overall cohort. Depending on sample availability and IgG yield, some pharyngitis samples were used across multiple experiments shown in Figures 3–6, whereas others were included only in specific assays. Binding assays were additionally repeated using five independent pharyngitis samples to confirm that the findings were representative of the broader cohort. In all mouse experiments, animals were randomized into cohorts of five by animal house staff.
Blinding	CHIVAS-M75 was an unblinded trial. The investigators on this study were not blinded to disease outcome. Before intranasal inoculation with S. pyogenes-IgG, mice undergoing the procedure were blinded by a researcher not directly involved in the experiment. Clinical scoring and bacterial burden (CFU) measurements were performed under blinded conditions, and mice were only unblinded after all data had been collected and analysed.

Reporting for specific materials, systems and methods

We require information from authors about some types of materials, experimental systems and methods used in many studies. Here, indicate whether each material, system or method listed is relevant to your study. If you are not sure if a list item applies to your research, read the appropriate section before selecting a response.

Materials & experimental systems

n/a	Involved in the study	
☐	☒	Antibodies
☒	☐	Eukaryotic cell lines
☒	☐	Palaeontology and archaeology
☐	☒	Animals and other organisms
☐	☒	Clinical data
☒	☐	Dual use research of concern
☒	☐	Plants

Methods

n/a	Involved in the study	
☒	☐	ChIP-seq
☐	☒	Flow cytometry
☒	☐	MRI-based neuroimaging

Antibodies

Antibodies used	Anti-human IgA (Invitrogen/Thermo Fisher, #A18781, polyclonal), used at 1:10,000<br>Anti-human IgG (H+L) (Invitrogen/Thermo Fisher, #31135, polyclonal), used at 1:2,000.<br>Anti-human IgG1 Fc (Invitrogen/Thermo Fisher, #A-10648, clone HP6069), used at 1:2,000.<br>Anti-human IgG2 (Invitrogen/Thermo Fisher, #MH1722, clone HP6014), used at 1:1,000.<br>Anti-human IgG3 hinge (Invitrogen/Thermo Fisher, #05-3620, clone HP6047), used at 1:1,000.<br>Anti-human IgG4 Fc (Invitrogen/Thermo Fisher, #A-10654, clone HP6025), used at 1:2,000.<br>Goat anti-mouse IgG (H+L)-HRP (Bio-Rad, #1706516, polyclonal), used at 1:500.<br>CD19 BUV395 (BioLegend, #302239, clone HIB19), used at 1:200.<br>CD21 FITC (BioLegend, #354910, clone Bu32), used at 1:200.<br>CD27 BV421 (BioLegend, #356417, clone M-T271), used at 1:400.<br>IgD PE/Dazzle 594 (BioLegend, #348239, clone IA6-2), used at 1:400.<br>IgM BV711 (BioLegend, #314539, clone MHM-88), used at 1:200.<br>IgG Fc BV510 (BioLegend, #410716, clone M1310G05), used at 1:200.<br>IgA PerCP-Vio700 (Miltenyi Biotec, #130-113-478, clone IS11-8E10), used at 1:300.<br>Anti-human IgG Fab secondary antibody PE (Invitrogen, #MA1-10377, clone 4A11), used at 1:200<br>Anti-mouse IgG1 PE/Dazzle 594 (BioLegend, #406628, clone RMG1-1), used at 1:200.
Validation	All antibodies used in this study were selected based on manufacturer validation data and previously published literature. Manufacturer datasheets included validation for ELISA and flow cytometry applications and provided recommended working concentrations.

For flow cytometry experiments, antibody concentrations were optimized empirically in human PBMC samples by titration to determine staining conditions that provided maximal signal-to-noise separation while minimizing background staining. Human TruStain FcX™ Fc receptor blocking reagent was included to reduce nonspecific Fc receptor-mediated staining.

For ELISA assays, HRP-conjugated secondary antibodies and human Ig isotype-specific antibodies were validated by the manufacturers for immunoassay applications and used according to recommended conditions. Antibodies used for S. pyogenes binding assays were validated by the manufacturers for immunoglobulin detection and flow cytometry applications.

Animals and other research organisms

Policy information about studies involving animals; ARRIVE guidelines recommended for reporting animal research, and Sex and Gender in Research

Laboratory animals	Female C.B-17/IcrHanHsd-Prkdcscid/Ozarc (SCID) mice (Mus musculus) aged 4–6 weeks were sourced from Ozgene, Western Australia
Wild animals	The study did not involve wild animals
Reporting on sex	Only female mice were used in this study due to their lower aggression and improved suitability for group housing.
Field-collected samples	The study did not involve samples collected from the field
Ethics oversight	All animal protocols were reviewed and approved by the Griffith University Animal Ethics Committee (GU-AEC) in accordance with the National Health and Medical Research Council (NHMRC) of Australia guidelines.

Note that full information on the approval of the study protocol must also be provided in the manuscript.

Clinical data

Policy information about clinical studies

All manuscripts should comply with the ICMJE guidelines for publication of clinical research and a completed CONSORT checklist must be included with all submissions.

Clinical trial registration	NCT03361163
Study protocol	Included in the supplementary appendix of "Osowicki, J et al. A controlled human infection model of Streptococcus pyogenes pharyngitis (CHIVAS-M75): an observational study. The Lancet Microbe, 2021.
Data collection	Participants challenged between July 10, 2018 (first participant, first dose) and Sep 2023, 2019 in Melbourne, Australia
Outcomes	For functional assay: bacterial-IgG binding (binding) and bacterial growth by culture (CFU).ELISA - optical density

Plants

Seed stocks	*Report on the source of all seed stocks or other plant material used. If applicable, state the seed stock centre and catalogue number. If plant specimens were collected from the field, describe the collection location, date and sampling procedures.*
Novel plant genotypes	*Describe the methods by which all novel plant genotypes were produced. This includes those generated by transgenic approaches, gene editing, chemical/radiation-based mutagenesis and hybridization. For transgenic lines, describe the transformation method, the number of independent lines analyzed and the generation upon which experiments were performed. For gene-edited lines, describe the editor used, the endogenous sequence targeted for editing, the targeting guide RNA sequence (if applicable) and how the editor was applied.*
Authentication	*Describe any authentication procedures for each seed stock used or novel genotype generated. Describe any experiments used to assess the effect of a mutation and, where applicable, how potential secondary effects (e.g. second site T-DNA insertions, mosiacism, off-target gene editing) were examined.*

Flow Cytometry

Plots

Confirm that:

- [x] The axis labels state the marker and fluorochrome used (e.g. CD4-FITC).
- [x] The axis scales are clearly visible. Include numbers along axes only for bottom left plot of group (a 'group' is an analysis of identical markers).
- [x] All plots are contour plots with outliers or pseudocolor plots.
- [x] A numerical value for number of cells or percentage (with statistics) is provided.

Methodology

Sample preparation	PBMCs were collected using CPT tubes (BD Bioscience) and stored in nitrogen. Frozen mononuclear PBMCs were thawed at 37ºC in thawing media (RPMI + 10% FCS), then washed and resuspended in flow buffer (PBS + 2%FCS + 2 mM EDTA).
Instrument	BD LSRFortessa (5-laser)
Software	BD FACSDiva™ software
Cell population abundance	N/A
Gating strategy	PBMC analysis: Lymphocytes were first gated based on forward and side scatter (FSC/SSC). Viable B cells were identified as $CD19^+$ (BUV395) cells after exclusion of debris and non-B cells. From the viable B-cell population, memory B cells were defined as $CD27^+CD21^+$ cells. Memory B cells were further subdivided based on IgD and IgM expression to identify IgD^-IgM^- memory B cells. This population was then analysed for class-switched immunoglobulin expression, distinguishing IgA^+ and IgG^+ memory B cells using IgA–PerCP-Vio700 and IgG–BV510 staining. Tetramer-positive cells were defined within the IgG^+ or IgA^+ populations based on dual APC^+PE^+ staining.<br><br>IgG-S. pyogenes binding analysis: S. pyogenes bacteria were gated based on FSC/SSC characteristics and doublets/aggregates were excluded using FSC-A/FSC-H gating. IgG binding was detected using a fluorescent anti-human IgG-PE or anti-mouse IgG1-PE/Dazzle 594 secondary antibody. Binding was quantified as the median fluorescence intensity (MFI) of IgG within the gated bacterial population.

☒ Tick this box to confirm that a figure exemplifying the gating strategy is provided in the Supplementary Information.
