# Supplementary material for: Subclinical exposure to Streptococcus pyogenes drives the development of long-lived homologous immunity
Source: Nat Commun. 2026 Aug 12;17:7850. doi: 10.1038/s41467-026-76176-1 (PMC13470482; doi:10.1038/s41467-026-76176-1)
Supplement: Supplementary file 3 [file 41467_2026_76176_MOESM3_ESM.pdf]

nature portfolio

Peer Review File

Subclinical exposure to Streptococcus pyogenes drives the development of long-lived homologous immunity

Corresponding Author: Professor Manisha Pandey

This file contains all reviewer reports in order by version, followed by all author rebuttals in order by version.

Version 0:

Reviewer comments:

Reviewer #1

(Remarks to the Author)
This manuscript characterizes the immune responses in humans that were challenged with live Streptococcus pyogenes M75 via pharyngeal inoculation. Both individuals that developed active pharyngitis (n=13) but also the group that were challenged but did not develop disease to represent subclinical exposure (n=5), were characterized. The research demonstrates that from the human challenge experiments, neither group invoked an antibody or memory B cell response to the p*17 peptide that is being tested as a vaccine. However, patients with symptomatic disease did develop memory B cells to the hypervariable region of M75, with an increase in IgG and IgA when the cohort was taken together. Mass spectrometry analyses of the antibodies demonstrated altered glycosylation of the Fc region of IgG to the M75 protein although what this means is quite speculative. The immunodeficient SCID mice experiments do support that the antibodies induced protection from S. pyogenes infection in vivo. Overall, the major finding that immunity is triggered to asymptomatic exposure represents a very important advance in our understanding of immune responses to S. pyogenes.

My suggestions are as follows:
In Figure 1E and 1F, there does seem to be some binding from the naïve serum against the M75 strain – does this strain express and IgG binding protein to explain this? This can also be seen in Figure 6B. This doesn't detract from the manuscript but perhaps the authors could comment on this.
Line 242 states that M75-specific IgG levels were very low in pre-challenge participants although in Figure 3C there does seem to be antibodies in some of the patients. I would consider revising this statement.
In Figure 5D, there appears to be some missing data for panel 3 (% of afucosylation – check spelling here) or are those data points all identical?
The data in Figure 6 are quite interesting where total IgG clearly binds to the M75 strain, but not the M1 strain, suggesting that the responses were dominant against the M protein but not to other shared surface proteins/antigens. I think this point could be expanded on. Furthermore, is this really a pneumonia model of nasal infection since you are recovering 106 CFUs from the lungs? This would suggest antibodies from subclinical (asymptomatic) infections could be protective against subsequent severe infections, not just in preventing pharyngitis reoccurrence.
The use of the word "tetramer" may be confusing to readers as tetramers typically make use of tetramer MHC molecules plus peptide for assessing T cells.
Line 120: changed the acronym from M75-HVR-DT to M75-DT
Line 208: There was no significant difference in pre-challenge M75 specific B cells and antibodies between pharyngitis and non-pharyngitis group, but it does seem like the pharyngitis group might be trending to have higher IgG titres? So are anti-M antibodies not necessarily protective or could this just be a result of low n value in non-pharyngitis group
Line 558. Strain 5448 was not isolated in Australia. See PMID: 10816507
Reference 31 is now published.

Reviewer #2

(Remarks to the Author)
Summary
This study uses samples from a human challenge model of pharyngeal S. pyogenes infection to investigate whether the development of symptoms is necessary for triggering humoral and cellular immune responses. Also, the paper attempts to

address whether M75-specific antibodies from asymptomatic participants are different than symptomatic patients.

Major comment 1:
The main issue with this paper is that is very descriptive and lacks mechanistic data. While the paper properly addresses that asymptomatic carriage of S. pyogenes can lead to functional antibody responses against M75, the manuscript fails to provide mechanistic insights into why M75-IgGs from asymptomatic individuals are more potent than those from symptomatic patients.
First, the authors show that challenge with M75 S. pyogenes induced M75-specific serum antibodies (and memory B cell) in both symptomatic and asymptomatic participants. Then they move on to purify M75-specific IgGs in order to explain difference in clinical outcome, but proper experiments are lacking.

* M75-specific IgG from asymptomatic participants had an altered Fc glycosylation signature. As previously demonstrated for anti-viral responses, this altered Fc signature could indicate enhanced effector function, but this is not directly shown for GAS in the paper. In vitro experiments demonstrating Fc-mediated effector functions on M75 (IgG-mediated complement opsonization, FcyR-mediated phagocytosis, complement-dependent phagocytosis) are required to address this point.

*The in vivo study (Fig. 6D-F) lacks proper controls. Only the effect of IgGs from asymptomatic participants is tested, while it would be important to compare this with the IgG from symptomatic participants.
*Also, the in vivo data fail to demonstrate a role for the Fc-glycosylation (could Fc glycans be chemically removed or recombinantly engineered to demonstrate this is important)
*The only in vitro data with M75-IgGs are shown in Fig. 6A,B, in which binding of IgGs to bacteria is tested. Since binding of antibodies is mediated by Fab regions, potential Fc effector functions are not taken into account here.

*Functional differences between asymptomatic vs symptomatic IgGs could also be due to different subclass (symptomatic mainly have M75-IgG3) or Fab sequences. How is this corrected for?

Major comment 2:
What is the rationale behind assessing 13 symptomatic and (only?) 5 asymptomatic individuals.

Major comment 3:
While the study focuses on M75-specific IgG responses in human infection, the Results section begins with data of a murine model in which M75-HVR and p*17 specific humoral responses is investigated. The connection with the human study is unclear and the rationale for the mouse experiments (plus conclusion) are not explained.

Minor comment:
1. Abstract and Page 4: it is mentioned that IgGs are “bactericidal/ ‘kill’ S. pyogenes”. This is misleading since antibodies are not known to directly kill Gram-positive bacteria. Rephrase to make clear that this is indirect killing via activation of Fc-effector mechanisms.
2. Some paragraphs of the Results section are very difficult to read because of all the details between brackets. (for example, Fc glycosylation paragraphs)

Reviewer #3

(Remarks to the Author)
Reviewer Comments

Summary
This manuscript reports that asymptomatic (“subclinical”) S. pyogenes exposure in a human challenge model induces strain-specific humoral responses, including increases in M75-HVR–specific memory B cells and serum IgG/IgA, antigen-specific Fc-glycosylation changes, and protection in a passive-transfer mouse model. The authors contrast these with minimal responses to the conserved p*17 epitope.
The study leverages a rare, well-controlled human challenge cohort and combines serology, memory B-cell analysis, glycoprofiling, and functional in vivo data. However, several aspects of the interpretation, methodology, and statistical analysis require clarification or revision before the claims can be supported.

Major Comments

1. Interpretation of binding data in Fig. 6 (“no binding to M1”)
The statement that there is “no binding to M1” (Fig. 6B, lines ~333–334) is not supported by the presented data and is highly unexpected. The literature shows that many individuals have natural IgG that binds S. pyogenes, including non-type-specific antibodies, even before infection.
• MFI baselines in Fig. 6 are >1,000, suggesting baseline binding is present, but without negative and positive controls and gating plots, it is impossible to tell. All that can be said now is that the baseline binding is largely unchanged against M1.
• Recommendation: Include full gating plots and positive and negative controls, interpret changes relative to these, provide dilution curves (1–1,000 µg/mL IgG) for M75 and M1, and also include additional S. pyogenes strains.
• The rationale for overnight antibody–bacteria incubation (see Methods) should be explained. Binding normally reaches equilibrium within 30 min; extended incubation risks IdeS/SpeB cleavage and Fc-binding protein interference.

• State whether the anti-human IgG Fab secondary detects all IgG subclasses equally.
• The de Neergaard et al. 2022 study (Microbiol. Spectr. 10, e02486-22). provides relevant recent evidence challenging strict type-specific opsonic immunity, and should be discussed to contextualize these findings.

2. Heavy reliance on synthetic peptides
Most binding and MBC data (Figs. 2–3, 5) are based on linear peptides from M75 or M1. These do not reproduce the coiled-coil conformation or native epitope presentation of M proteins on the bacterial surface.
• Recommendation: Discuss this limitation explicitly and, if possible, validate with whole-bacterium or whole-protein assays.

3. Fc-binding proteins in challenge strains
Several S. pyogenes strains express Fc-binding proteins (M protein Fc domain, protein H), which can alter apparent Fab-mediated binding.
• Recommendation: Report whether the M75 and M1 strains have Fc-binding capacity (they should) and whether assays controlled for these (e.g., $F(ab')_2$ fragments).

4. Effect size vs. statistical significance in Figs. 2–3
MBC frequencies are very low (M75: ~0.5 → 0.75%; p*17: ~1–2%) with modest changes. In Fig. 3, ANOVA detects significance despite small magnitude; in Fig. 2, a larger 1–6 mo change is dismissed as “no effect.” ELISA IgG/IgA changes are similarly small.
• Recommendation: Present effect sizes with 95% CIs alongside P-values, and discuss biological as well as statistical significance. Present plots with the same Y-axis scaling when presenting data.

5. Opsonization details for intranasal challenge
The in vivo challenge lacks key opsonization details (IgG concentration, incubation time/temp, bacterial growth phase, viability).
• Recommendation: Provide complete protocol and controls

6. Statistical inconsistencies and 90% CI use
Legends (e.g., Fig. 6: ANOVA/Bonferroni) differ from Methods (Mann–Whitney, 90% CI). Use of 90% CI is unusual without strong justification.
• Recommendation: Standardize tests, clarify one-tailed vs. two-tailed tests; ensure analysis descriptions are consistent, include effect sizes with 95% CIs alongside p-values, and ensure Methods match legends.

Version 1:

Reviewer comments:

Reviewer #1

(Remarks to the Author)
I was pleased to see the detailed rebuttal and the inclusion of new experimental data such as the addition M protein serotypes. I have no further suggestions.

Reviewer #2

(Remarks to the Author)
The authors properly addressed the concerns raised by the referees. I have no further comments

Reviewer #3

(Remarks to the Author)
I'm happy with the revisions made.

Open Access This Peer Review File is licensed under a Creative Commons Attribution 4.0 International License, which permits use, sharing, adaptation, distribution and reproduction in any medium or format, as long as you give appropriate credit to the original author(s) and the source, provide a link to the Creative Commons license, and indicate if changes were made.
In cases where reviewers are anonymous, credit should be given to 'Anonymous Referee' and the source.
The images or other third party material in this Peer Review File are included in the article's Creative Commons license, unless indicated otherwise in a credit line to the material. If material is not included in the article's Creative Commons license and your intended use is not permitted by statutory regulation or exceeds the permitted use, you will need to obtain permission directly from the copyright holder.
To view a copy of this license, visit https://creativecommons.org/licenses/by/4.0/

REVIEWER COMMENTS

We thank the reviewer for their constructive and insightful comments, which have helped to improve the clarity and quality of the manuscript. We have carefully addressed each point raised and revised the text accordingly.

Reviewer #1 (Remarks to the Author):

This manuscript characterizes the immune responses in humans that were challenged with live Streptococcus pyogenes M75 via pharyngeal inoculation. Both individuals that developed active pharyngitis (n=13) but also the group that were challenged but did not develop disease to represent subclinical exposure (n=5), were characterized. The research demonstrates that from the human challenge experiments, neither group invoked an antibody or memory B cell response to the p*17 peptide that is being tested as a vaccine. However, patients with symptomatic disease did develop memory B cells to the hypervariable region of M75, with an increase in IgG and IgA when the cohort was taken together. Mass spectrometry analyses of the antibodies demonstrated altered glycosylation of the Fc region of IgG to the M75 protein although what this means is quite speculative. The immunodeficient SCID mice experiments do support that the antibodies induced protection from S. pyogenes infection in vivo. Overall, the major finding that immunity is triggered to asymptomatic exposure represents a very important advance in our understanding of immune responses to S. pyogenes.

My suggestions are as follows:

In Figure 1E and 1F, there does seem to be some binding from the naïve serum against the M75 strain – does this strain express and IgG binding protein to explain this? This can also be seen in Figure 6B. This doesn't detract from the manuscript but perhaps the authors could comment on this.

Thank you for bringing attention to this point. The M protein, M-related protein (Mrp), and M-like protein (Enn) expressed by the M75 strain are all predicted to bind IgG in a non–antigen-specific, Fc-mediated manner. Specifically, the M75 M protein has predicted IgG-binding activity (PMID: 24799598), all Mrp proteins have been shown to bind IgG (PMID: 38176650), and while Enn proteins display greater variability, the majority retain IgG-binding capacity (PMID: 33630944). Although we have reduced the likelihood of nonspecific binding by adding skim milk to our assays, it is possible that some degree of nonspecific binding is still observed.

Line 242 states that M75-specific IgG levels were very low in pre-challenge participants although in Figure 3C there does seem to be antibodies in some of the patients. I would consider revising this statement.

We have revised the statement above to address the reviewer's comment and clarify that individuals with high pre-challenge levels of type-specific anti-M75 IgG were excluded from the CHIVAS-M75 trial, although low levels of M75-specific IgG remained detectable in some participants at pre-challenge (page 8/line 197-200).

In Figure 5D, there appears to be some missing data for panel 3 (% of afucosylation – check spelling here) or are those data points all identical?

The points are identical, each with a value of 0.

The data in Figure 6 are quite interesting where total IgG clearly binds to the M75 strain, but not the M1 strain, suggesting that the responses were dominant against the M protein but not to other shared surface proteins/antigens. I think this point could be expanded on.

Thank you for your suggestion. We have expanded this analysis to test IgG binding against additional bacterial strains representing different emm tpes, including M12, M59, and M89 (Fig. 5C). Although some participants had pre-existing IgG antibodies, there were no significant differences in binding following challenge with M75 *S. pyogenes*. In accordance with the reviewer's comments, we have updated the discussion section to incorporate the information that an increase in serum binding to the M75 strain but not to heterologous strains indicates that the responses were dominant against the M-protein but not to other shared surface proteins/antigens (page 18/line 431-434).

Furthermore, is this really a pneumonia model of nasal infection since you are recovering 106 CFUs from the lungs? This would suggest antibodies from subclinical (asymptomatic) infections could be protective against subsequent severe infections, not just in preventing pharyngitis reoccurrence.

This is a murine model of *S. pyogenes* respiratory tract infection (page 4/line 106), which can be used for both pharyngeal infection studies and more severe diseases. The data indeed demonstrate that antibodies from subclinical infections can confer protection against subsequent severe/invasive infections. This information has been added at the end of the last results section (page 13/line 311-312).

The use of the word "tetramer" may be confusing to readers as tetramers typically make use of tetramer MHC molecules plus peptide for assessing T cells.

To our knowledge, B cell tetramers are defined in the same way as peptide–MHC tetramers for T cells. They both consist of four biotinylated ligands (B cell epitope or T cell peptide–MHC complexes) bound to streptavidin, forming a tetrameric probe that increases avidity for lymphocyte receptors. Please refer to PMID: 38243093 for more information.

Line 120: changed the acronym from M75-HVR-DT to M75-DT

Thank you for pointing this out. The acronym has now been corrected (page 5/lines 117, 122 and 124).

Line 208: There was no significant difference in pre-challenge M75-specific B cells and antibodies between pharyngitis and non-pharyngitis groups, but it does seem like the pharyngitis group might be trending to have higher IgG titres? So are anti-M antibodies not necessarily protective or could this just be a result of low n value in non-pharyngitis group

We believe this observation is likely due to the smaller number of samples analysed in the non-pharyngitis group. As noted in the Discussion section, a limitation of our study is the relatively small cohort size, particularly the limited number of asymptomatic participants. Importantly, pre-challenge antibody levels were comparable between groups for both M75-specific IgG (mean pharyngitis pre-challenge = 0.3273 ± 0.0388; non-pharyngitis pre-challenge = 0.2801 ± 0.0794; Fig. 2C) and IgG3 (mean pharyngitis pre-challenge = 0.1063 ± 0.0110; non-pharyngitis pre-challenge = 0.1129 ± 0.0211; Supp. Fig. 2F).

Line 558. Strain 5448 was not isolated in Australia. See PMID: 10816507.

Thank you for bringing this to our attention. The sentence in the Materials and Methods section has been updated to read *"…isolated from a case of invasive infection*," and the appropriate reference has now been included in the text (page 21/line 514).

Reference 31 is now published.

The medRxiv reference has now been replaced with the Nature Medicine reference.

Reviewer #2 (Remarks to the Author):

Summary

This study uses samples from a human challenge model of pharyngeal S. pyogenes infection to investigate whether the development of symptoms is necessary for triggering humoral and cellular immune responses. Also, the paper attempts to address whether M75-specific antibodies from asymptomatic participants are different than symptomatic patients.

Major comment 1:

The main issue with this paper is that is very descriptive and lacks mechanistic data. While the paper properly addresses that asymptomatic carriage of S. pyogenes can lead to functional antibody responses against M75, the manuscript fails to provide mechanistic insights into why M75-IgGs from asymptomatic individuals are more potent than those from symptomatic patients.

We thank the reviewer for their comment. As we have made clear in the revised version of the manuscript, our data do not indicate that M75-specific IgG from asymptomatic individuals are more potent than those from symptomatic participants. We apologise for any lack of clarity in the original text.

We observed changes in Fc glycosylation of M75-specific IgG following pharyngeal challenge only in the serum of asymptomatic participants. However, functional assessment of purified serum IgG using both *in vitro* and *in vivo* assays demonstrates that post-challenge IgG show an equally enhanced capacity to bind M75 *S. pyogenes* (Figure 5B) and to reduce colonization of the respiratory mucosa and organs, with concomitant improvements in clinical scores, regardless of clinical outcome (Figure 6 B and C). These functional effects were observed for post-challenge IgG overall and were not intended to imply superior potency of antibodies derived from asymptomatic individuals compared with those from symptomatic participants. To avoid misinterpretation, we have expanded the Results (page 13/ line 308-309) and Discussion to explicitly clarify that (page 16/line 393-396).

Additionally, a difference in tissue- or site-specific protective efficacy was observed. IgG from asymptomatic participants appeared to confer greater protection at mucosal sites, whereas IgG from symptomatic participants demonstrated higher efficacy at systemic sites. These differences may reflect qualitative differences in Fc-mediated function. In asymptomatic participants, Fc remodelling may favour interactions with Fcγ receptors and complement pathways that support efficient opsonisation and clearance at mucosal surfaces, whereas antibodies induced during symptomatic infection may be more effective in controlling bacterial burden within deeper tissues. However, in the absence of targeted functional and mechanistic analyses, the pathways driving these differential protective effects cannot be definitively

determined. This information has been added in the results (page 13/line 305-309) and discussion sections (page 16/line 396-402).

First, the authors show that challenge with M75 S. pyogenes induced M75-specific serum antibodies (and memory B cell) in both symptomatic and asymptomatic participants. Then they move on to purify M75-specific IgGs in order to explain difference in clinical outcome, but proper experiments are lacking.

* M75-specific IgG from asymptomatic participants had an altered Fc glycosylation signature. As previously demonstrated for anti-viral responses, this altered Fc signature could indicate enhanced effector function, but this is not directly shown for GAS in the paper. In vitro experiments demonstrating Fc-mediated effector functions on M75 (IgG-mediated complement opsonization, FcγR-mediated phagocytosis, complement-dependent phagocytosis) are required to address this point.

The reviewer raises an important point regarding the functional relevance of the altered Fc glycosylation signature observed in M75-specific IgG from asymptomatic participants. Although sample availability from this group is limited, we selected serum IgG from a representative asymptomatic participant to assess the impact of pharyngeal challenge on Fc-mediated effector function using an opsonophagocytic killing assay (OPKA). In this assay, purified pre- and post-challenge IgG were incubated with M75 *S. pyogenes* in the presence of differentiated HL-60 cells (Figure 1 for reviewer). Results demonstrate increased opsonic titres, defined as the highest dilution of test (6mo post) sera that shows lower CFU count than the mean of control (pre) sera, for post-challenge IgG compared with pre-challenge IgG. Our results show enhanced opsonophagocytic killing capacity following challenge. These findings suggest that, at least in some cases, the observed changes in M75-specific IgG Fc glycosylation may be associated with improved Fc-dependent effector function. However, as these experiments were performed using total IgG rather than affinity-purified M75-specific IgG, we cannot exclude the contribution of antibodies targeting additional bacterial antigens to the increased opsonization observed. Nevertheless, the increase in serum IgG binding to the M75 strain, but not to heterologous strains (Fig. 5), suggests that the response was predominantly directed against the M protein. We have added this limitation to the Discussion (page 18/ line 431-437).

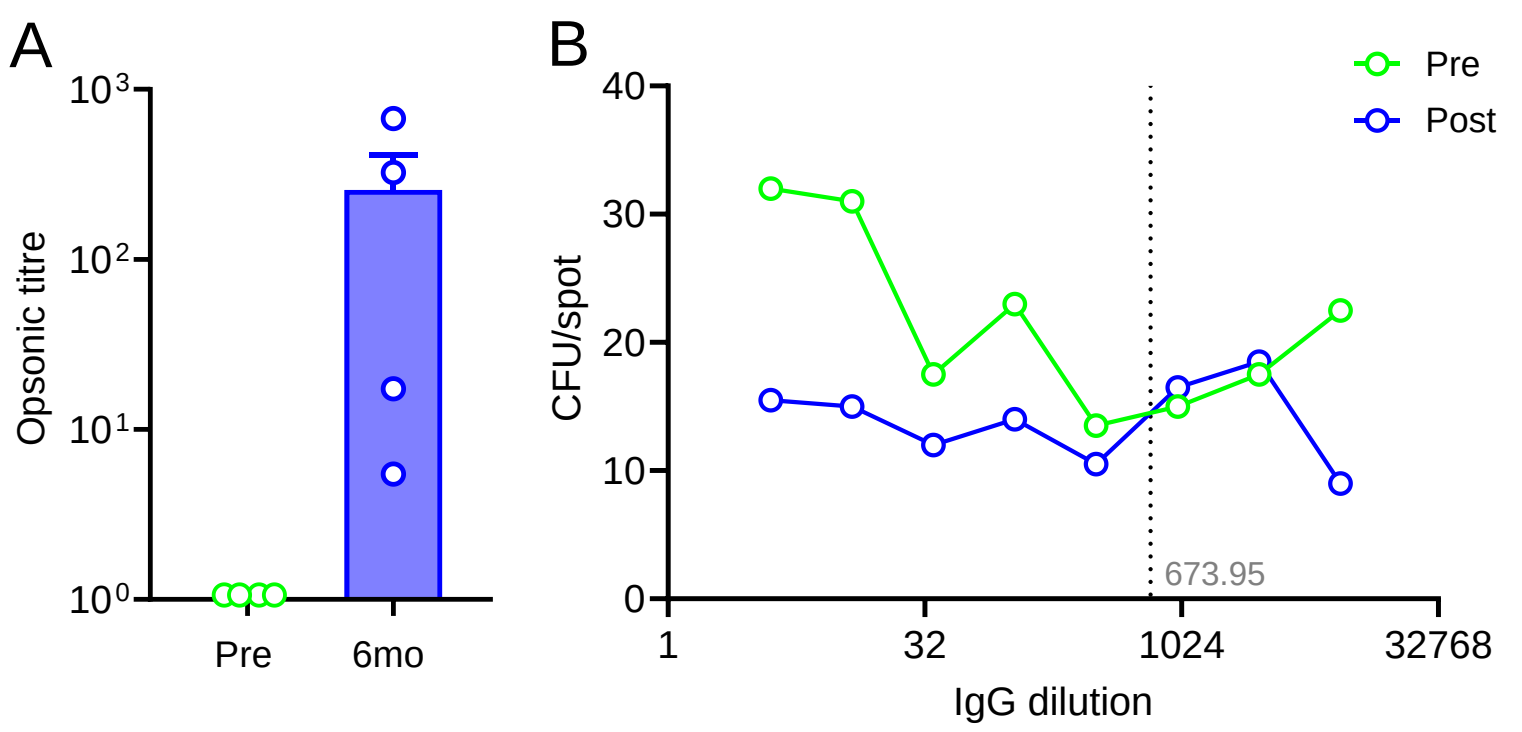

Figure 1 for reviewer. (A) Comparison of pre- and post-challenge opsonic titres of IgG purified from a single asymptomatic participant across four independent experiments. Data are

presented as mean ± SEM. (B) Representative plot showing mean CFU counts at each dilution, averaged across the four experiments. Pre-challenge samples are presented as green circles and post-challenge samples as blue circles. The opsonic titre is denoted by the dotted line.

*The in vivo study (Fig. 6D-F) lacks proper controls. Only the effect of IgGs from asymptomatic participants is tested, while it would be important to compare this with the IgG from symptomatic participants.

The authors agree with the reviewer's comment and have therefore performed an additional *in vivo* study to directly assess the effect of IgG derived from symptomatic participants, both pre- and post-challenge. These new data have been added as Figure 6B. Consistent with the findings observed for IgG from asymptomatic participants, post-challenge IgG from symptomatic participants also significantly reduced *S. pyogenes* colonization of the respiratory mucosa and organs compared with pre-challenge IgG. This comparison confirms that the protective effect observed *in vivo* is associated with post-challenge IgG irrespective of clinical outcome and is not restricted to antibodies derived from asymptomatic individuals.

*Also, the in vivo data fail to demonstrate a role for the Fc-glycosylation (could Fc glycans be chemically removed or recombinantly engineered to demonstrate this is important)

We thank the reviewer for this insightful suggestion. We agree that approaches such as enzymatic removal or recombinant engineering of Fc glycans would be valuable to directly demonstrate the functional contribution of Fc glycosylation in vivo. However, we are limited by the amount of serum and purified IgG available from asymptomatic participants, which precludes repeating the *in vivo* experiments with additional glyco-engineered IgG preparations. Importantly, the most pronounced glycan changes following challenge were confined to M75-specific IgG, which can only be isolated in very small quantities, further limiting the feasibility of such approaches. In addition, even if sufficient IgG were available, complete enzymatic removal or modification of Fc glycans would not accurately recapitulate the pre-challenge state, as we observed concurrent increases in both agalactosylation and sialylation rather than a uniform shift in glycan composition. Despite these constraints, our *in vivo* data using a mouse model that lacks adaptive immunity (Fig. 6), together with the glycomics analyses presented (Fig. 3 and 4), support a role for Fc glycosylation in modulating antibody function. To address this limitation, we have added this into the Discussion section (page 17/ line 422-426).

*The only in vitro data with M75-IgGs are shown in Fig. 6A,B, in which binding of IgGs to bacteria is tested. Since binding of antibodies is mediated by Fab regions, potential Fc effector functions are not taken into account here.

The newly generated OPKA data presented in Figure 1 of this rebuttal indicate that, in at least some cases, increased antibody binding is associated with enhanced Fc-mediated effector functions following challenge. Furthermore, the *in vivo* protection observed in SCID mice, which lack adaptive immunity, is most consistent with Fc-dependent mechanisms, as bacterial clearance in this model depends on interactions between passively transferred IgG and innate immune effectors, including neutrophils.

*Functional differences between asymptomatic vs symptomatic IgGs could also be due to different subclass (symptomatic mainly have M75-IgG3) or Fab sequences. How is this corrected for?

We thank the reviewer for highlighting this important consideration. As shown in Supp. Fig. 2B, overall M75-specific IgG3 levels were similar in both symptomatic and asymptomatic participants. Importantly, we are not claiming functional differences between symptomatic and asymptomatic IgG, as IgG from both groups is capable of binding to M75 *S. pyogenes* (Figure 5B) and reducing bacterial dissemination *in vivo* (Figure 6). Thus, any apparent functional difference between both groups is likely due to the smaller sample size of the non-pharyngitis group (n = 5) relative to the pharyngitis group (n = 13). While we did not perform Fab sequencing to assess an association between clinical outcomes and IgG antibody repertoire, we cannot exclude the possibility that differences in clinical outcomes were also influenced by the generation of distinct antibody clones following challenge. We have acknowledged this limitation and included a corresponding statement in the Discussion (page 17-18/ line 426-428).

Major comment 2:

What is the rationale behind assessing 13 symptomatic and (only?) 5 asymptomatic individuals.

This is a limitation with the CHIVAS-M75 study samples. The study was designed to induce acute pharyngitis in approximately 60% of healthy adult volunteers following pharyngeal swab challenge (PMID: 35544165). In practice, however, more than 80% of participants developed pharyngitis, resulting in a relatively small asymptomatic cohort. Consequently, only a limited number of asymptomatic samples were available and could be included in the analyses presented in this manuscript.

Major comment 3:

While the study focuses on M75-specific IgG responses in human infection, the Results section begins with data of a murine model in which M75-HVR and p*17 specific humoral responses is investigated. The connection with the human study is unclear and the rationale for the mouse experiments (plus conclusion) are not explained.

To our knowledge, this is the first study to characterize immune responses to the M75-HVR. As this peptide was used in both tetramer staining and ELISA, we validated it to confirm specificity for the hypervariable region and to exclude recognition of conserved M protein epitopes. We agree with the reviewer that the connection to the human study was not sufficiently clear and have therefore moved these data to the Supplemental Figures (Supplementary Figure 1).

Minor comment:

1. Abstract and Page 4: it is mentioned that IgGs are “bactericidal/ ‘kill’ S. pyogenes”. This is misleading since antibodies are not known to directly kill Gram-positive bacteria. Rephrase to make clear that this is indirect killing via activation of Fc-effector mechanisms.

We thank the reviewer for this point and have revised the text to clarify that IgG clears bacteria indirectly:

Abstract-line 46: The original sentence “*...and were bactericidal when transferred into a murine model of pharyngeal infection*.” has been rephrased to “…*and conferred protection when transferred into a murine model of pharyngeal infection*.”

Page 4/line 86-87: The original sentence "*We previously showed that antibodies to the conserved C-repeat region can kill the S. pyogenes"* has been rephrased to *"We previously demonstrated that antibodies to the conserved C-repeat region promote immune-mediated killing of S. pyogenes*"

2. Some paragraphs of the Results section are very difficult to read because of all the details between brackets. (for example, Fc glycosylation paragraphs)

Thank you for raising this concern. We have carefully revised the relevant sections of the Results, particularly those describing Fc glycosylation (pages 8 and 9), to reduce the density of bracketed information. Where possible, we have incorporated key details into the main text.

Reviewer #3 (Remarks to the Author):

Reviewer Comments

Summary

This manuscript reports that asymptomatic ("subclinical") S. pyogenes exposure in a human challenge model induces strain-specific humoral responses, including increases in M75-HVR–specific memory B cells and serum IgG/IgA, antigen-specific Fc-glycosylation changes, and protection in a passive-transfer mouse model. The authors contrast these with minimal responses to the conserved p*17 epitope. The study leverages a rare, well-controlled human challenge cohort and combines serology, memory B-cell analysis, glycoprofiling, and functional in vivo data. However, several aspects of the interpretation, methodology, and statistical analysis require clarification or revision before the claims can be supported.

Major Comments

1. Interpretation of binding data in Fig. 6 ("no binding to M1")

The statement that there is "no binding to M1" (Fig. 6B, lines ~333–334) is not supported by the presented data and is highly unexpected. The literature shows that many individuals have natural IgG that binds S. pyogenes, including non-type-specific antibodies, even before infection.

We thank the reviewer for raising this important point. We performed additional binding assays to assess the binding of 5 extra symptomatic donors to M1 and also to M59, M12 and M89. These data are included in the new Figure 5C. We show high baseline binding of IgG from some participants to these strains (>2000 MFI), supporting the presence of pre-existing antibodies. We have revised the statement to indicate that (page 11/ line 271-273).

• MFI baselines in Fig. 6 are >1,000, suggesting baseline binding is present, but without negative and positive controls and gating plots, it is impossible to tell. All that can be said now is that the baseline binding is largely unchanged against M1.

• Recommendation:

-Include full gating plots and positive and negative controls, interpret changes relative to these,

To address this comment, negative controls were generated by absorbing normal human IgG against M1 and M75 *S. pyogenes*. A new binding assay was performed using IgG from five symptomatic participants (not previously tested) and two asymptomatic participants (repeated

from the previous experiment), with binding interpreted relative to these negative controls. Intravenous immunoglobulin (IVIg) was included as a positive control to confirm assay performance. The data shown in the revised Figures 5B and C represent a combination of both experiments. For M1, the negative control MFI was 660, and pre-challenge IgG MFIs ranged from 104 to 1430, consistent with binding that was comparable to, or only marginally above, background levels (Fig. 5C i). For clarity, the flow histograms were labelled to show the individual groups. *S. pyogenes* bacteria were gated based on FSC/SSC characteristics (Figure 5A) and doublets/aggregates were excluded using FSC-A/FSC-H gating. IgG binding was detected using a fluorescent anti-human IgG-PE or anti-mouse IgG1-PE/Dazzle 594 secondary antibody. We have incorporated the interpretation of these findings into the revised manuscript (pages 10 and 11/ line 253-273).

-Provide dilution curves (1–1,000 µg/mL IgG) for M75 and M1,

Due to the limited availability of purified IgG for this analysis, particularly from asymptomatic participants, we have only selected two participants with pharyngitis and one asymptomatic to provide the dilution curves (Figure 1 for reviewer). Consistently, post-challenge IgG showed increased, dose-dependent binding to M75 compared with pre-challenge IgG, particularly in participants with pharyngitis, while binding to M1 remained low at both time points. When testing multiple concentrations of purified IgG for both M1 and M75, we observed a bell-shaped binding curve, which is consistent with the classical prozone/hook effect, where excessive antibody levels can interfere with immune complex formation and reduce measurable signal at very high concentrations. The optimal concentration for M75 was 10 µg/mL, as this was the concentration at which maximal binding was observed based on the dilution curves. In contrast, for M1, the highest binding generally occurred at 100 µg/mL (except for C68), which may reflect reduced specificity. Overall, these data indicate that infection induces a predominantly strain-specific IgG response with limited heterologous reactivity.

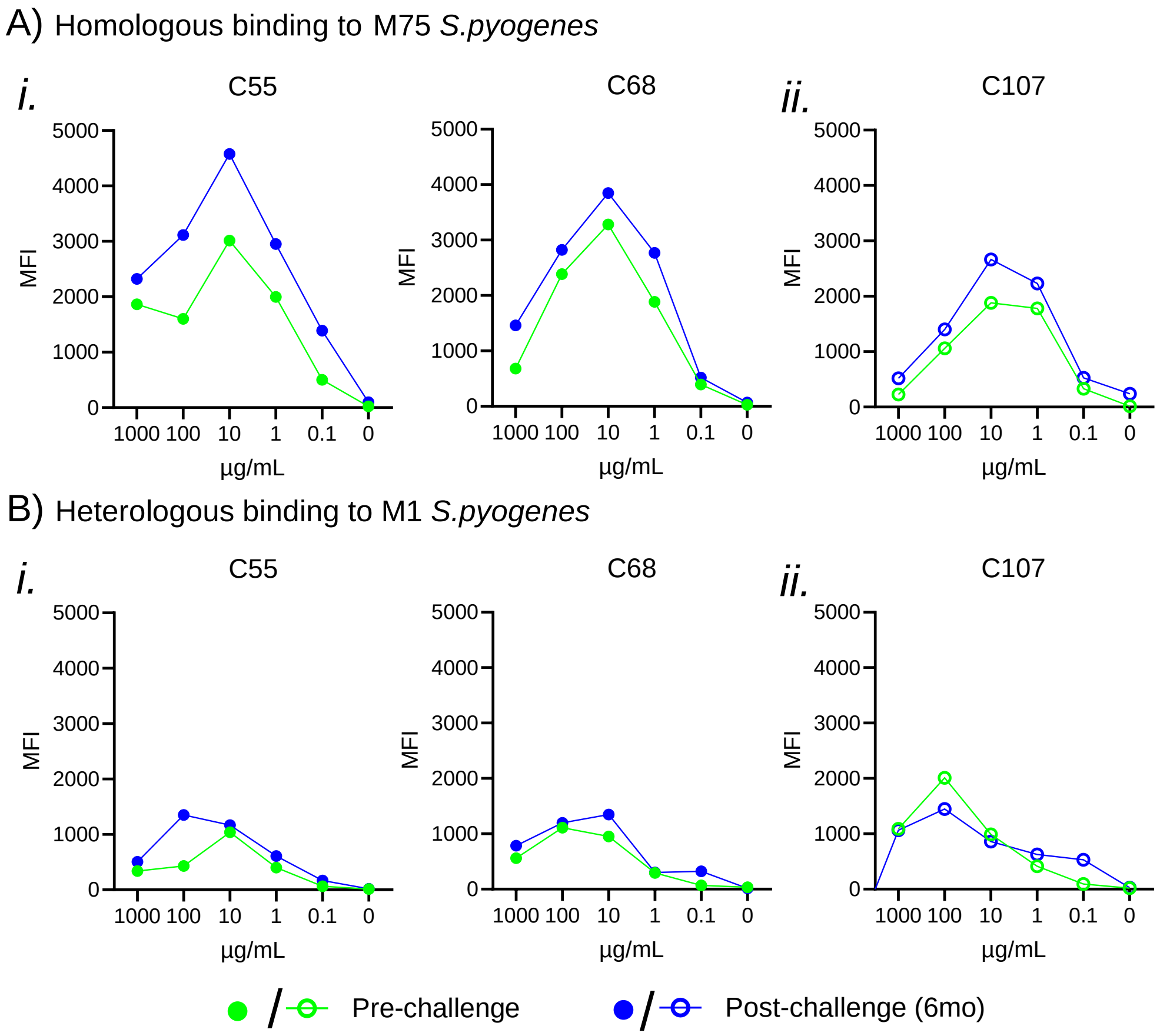

Figure 1 for the reviewer. IgG binding to homologous and heterologous S. pyogenes across a range of antibody concentrations. Representative flow cytometry–based binding curves showing IgG binding to homologous M75 and heterologous M1 S. pyogenes at increasing antibody concentrations. Green lines represent IgG purified pre-challenge, and blue lines represent IgG purified at 6 months post-challenge. Data are shown as mean fluorescence intensity (MFI).

-Include additional *S. pyogenes* strains.

Binding data to M12, M59 and M89 have been added to the revised manuscript as Figure 5C, *ii-iv*.

• The rationale for overnight antibody–bacteria incubation (see Methods) should be explained. Binding normally reaches equilibrium within 30 min; extended incubation risks IdeS/SpeB cleavage and Fc-binding protein interference.

Overnight incubation was selected based on preliminary optimisation experiments using serum from mice immunised with P*17-K4S2-DT/Alum or naïve controls. Serum from immunised mice showed improved binding to *S. pyogenes* following overnight incubation (at 4 °C) compared with shorter incubations (45 minutes at room temperature; see Figure 2 for reviewer). Overnight incubations were performed at 4 °C to minimise bacterial metabolic activity and reduce the potential for IgG cleavage by IdeS or SpeB.

To minimise potential Fc-mediated interactions with bacterial Fc-binding proteins, recombinant human Fc or skim milk was included as a blocking reagent. Comparable binding

profiles were observed with both blocking strategies, with binding of vaccinated mouse serum to *S. pyogenes* consistently higher following overnight incubation compared with 45-minute incubation (see Figure 2 for Reviewer). In contrast, binding of naïve serum remained uniformly low and did not differ between incubation times under any condition. These findings support the interpretation that the enhanced bacteria–antibody interactions observed with prolonged incubation are predominantly mediated through the IgG Fab region rather than Fc-dependent binding.

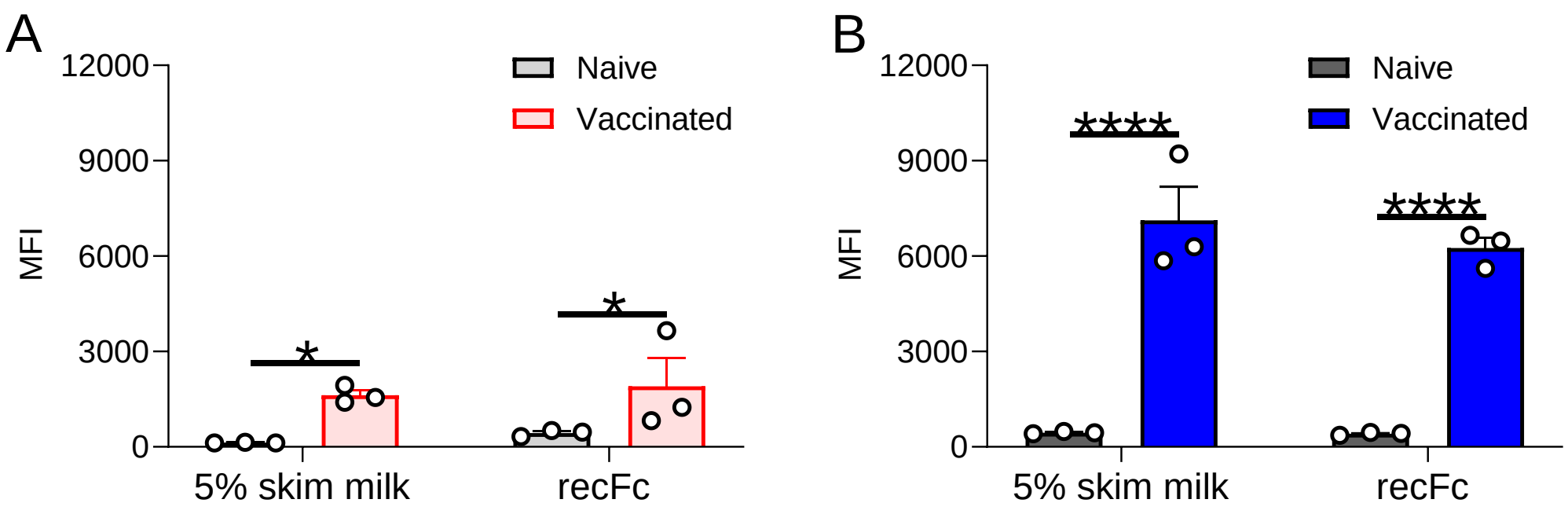

*Figure 2 for reviewer. Effect of incubation time and blocking conditions on IgG-mediated binding to S. pyogenes. Binding of S. pyogenes to antibodies was assessed after (A) 45 min or (B) overnight incubation using different blocking agents: 5% skim milk OR recombinant Fc protein (recFc). Naïve and vaccinated sera were compared. Binding is expressed as median fluorescence intensity (MFI). Individual data points represent biological replicates, and bars show mean ± SEM. *P<0.05, ****P<0.001.*

• State whether the anti-human IgG Fab secondary detects all IgG subclasses equally.

The mouse anti-human IgG Fab secondary antibody (MA110377) is designed to bind the Fab region, which is highly conserved across all human IgG subclasses (IgG1–4). Therefore, it should detect all IgG subclasses and avoid Fc-dependent biases, which can differ substantially between IgG subclasses.

• The de Neergaard et al. 2022 study (Microbiol. Spectr. 10, e02486-22). provides relevant recent evidence challenging strict type-specific opsonic immunity, and should be discussed to contextualize these findings.

Thanks for bringing this important work to our attention. We believe that the route of infection (invasive *versus* superficial infection) could differentially affect the breadth of immune responses. This is based on the present study and our previous publication that demonstrated strain-specific immunity following skin/superficial (PMID: 28027314) and upper respiratory tract (PMID: 38576622) *S. pyogenes* infection. We have now added this observation to the discussion (page 15/ line 356-362).

Most binding and MBC data (Figs. 2–3, 5) are based on linear peptides from M75 or M1. These do not reproduce the coiled-coil conformation or native epitope presentation of M proteins.

• Recommendation: Discuss this limitation explicitly and, if possible, validate with whole-bacterium or whole-protein assays.

The *S. pyogenes* binding to IgG data shown in Figure 5 was assessed using a live-bacterium assay, as recommended by the reviewer. The use of peptides for tetramer staining and ELISA allowed us to specifically identify antigen-specific B cell responses to the hypervariable regions (M75-HVR) and compare them with responses to conserved regions of the M protein (p*17/p145), which would not be possible using whole-bacterium or whole-protein assays.

Besides, M-protein HVR acts as a "nonideal" and irregular coiled-coil, which is more flexible or structurally irregular than the more conserved, rigid C-terminal part of the M protein, with high propensity for forming an α-helical coiled coil (PMID 21557065, 18323455).

4. Effect size vs. statistical significance in Figs. 2–3

MBC frequencies are very low (M75: ~0.5 → 0.75%; p*17: ~1–2%) with modest changes. In Fig. 3, ANOVA detects significance despite small magnitude; in Fig. 2, a larger 1–6 mo change is dismissed as "no effect." ELISA IgG/IgA changes are similarly small.

• Recommendation: Present effect sizes with 95% CIs alongside P-values, and discuss biological as well as statistical significance.

We agree with the reviewer that presenting effect sizes is an appropriate approach for reporting differences across this dataset, particularly given the unequal number of samples between the pharyngitis (n=13) and non-pharyngitis (n=5) groups. Accordingly, we have revised the manuscript to report the mean differences in tetramer and antibody responses post-challenge, along with 95% confidence intervals and corresponding P-values (pages 6 and 7). We have also expanded the discussion to address both the biological and statistical significance of these findings. (page 13/line 321-326).

Present plots with the same Y-axis scaling when presenting data.

We thank the reviewer for this suggestion. However, because serum IgA responses show lower O.D. values compared with IgG, using the same scale would make differences between pre- and post-challenge difficult for readers to discern. This is not unexpected, given that IgA constitutes only a small fraction of total serum immunoglobulin compared with IgG. In contrast, for the tetramer data we have used the same y-axis scaling, as the values are comparable between IgA^+ and IgG^+ cells.

5. Opsonization details for intranasal challenge

The in vivo challenge lacks key opsonization details (IgG concentration, incubation time/temp, bacterial growth phase, viability).

• Recommendation: Provide complete protocol and controls

We have matched the concentration of purified IgG cross all conditions analysed in the *in vivo* assays. For pre-incubation with *S. pyogenes*, 10 µL of IgG (30 mg/mL) was incubated with 5 µL of stationary phase live bacteria (OD_{600} = 0.5) for 45 minutes at room temperature. The detailed information has now been added to the methods section of the paper (page 22, line 354-355).

6. Statistical inconsistencies and 90% CI use

Legends (e.g., Fig. 6: ANOVA/Bonferroni) differ from Methods (Mann–Whitney, 90% CI). Use of 90% CI is unusual without a strong justification.

• Recommendation: Standardize tests, clarify one-tailed vs. two-tailed tests; ensure analysis descriptions are consistent, include effect sizes with 95% CIs alongside p-values, and ensure Methods match legends.

Thank you very much for highlighting this important point. We have corrected and standardized the analyses accordingly. In brief, one-way ANOVA with Tukey's post-hoc test was used for multiple paired comparisons, whereas a two-tailed paired t-test was applied when comparing only two groups. Effect sizes with 95% CIs alongside p-values are now included. An unpaired t-test was used for statistical analysis when comparing participants with and without pharyngitis (page 24, line 593-596).
